# Two Metalloproteases VdM35-1 and VdASPF2 from Verticillium dahliae Are Required for Fungal Pathogenicity, Stress Adaptation, and Activating Immune Response of Host

**DOI:** 10.1128/spectrum.02477-22

**Published:** 2022-10-12

**Authors:** Junyuan Lv, Jinglong Zhou, BaiYang Chang, Yihao Zhang, Zili Feng, Feng Wei, Lihong Zhao, Yalin Zhang, Hongjie Feng

**Affiliations:** a State Key Laboratory of Cotton Biology, Institute of Cotton Research of Chinese Academy of Agricultural Sciences, Anyang, Henan, China; b Western Agricultural Research Center of Chinese Academy of Agricultural Sciences, Changji, Xinjiang, China; Universidade de Sao Paulo

**Keywords:** *Verticillium dahliae*, metalloprotease, biological characteristics, virulence, stress response

## Abstract

Verticillium dahliae is a soilborne fungus that causes destructive vascular wilt diseases in a wide range of plant hosts. In this study, we identified two M35 family metalloproteinases: VdM35-1 and VdASPF2, and investigated their function *in vitro* and *in vivo*. The results showed that VdM35-1 and VdASPF2 were located in the cell membrane, as secreted proteins depended on signal peptide, and two histidine residues (H) induced cell death and activated plant immune response. VdM35-1 depended on membrane receptor proteins NbBAK1 and NbSOBIR1 in the process of inducing cell death, while VdASPF2 did not depend on them. The deletion of VdM35-1 and VdASPF2 led to the decrease of sporulation and the slow shortening of mycelial branch growth, and the spore morphology of VdM35-1-deficient strain became malformed. In addition, ΔVdM35-1 and ΔVdASPF2 showed more sensitive to osmotic stress, SDS, Congo red (CR), and high temperature. In terms of the utilization of carbon sources, the knockout mutants exhibited decreased utilization of carbon sources, and the growth rates on the medium containing sucrose, starch, and pectin were lower than the wild type strain, with significantly limited growth, especially on galactose-containing medium. Furthermore, ΔVdM35-1 and ΔVdASPF2 showed a significant reduction in pathogenicity. Collectively, these results suggested that VdM35-1 and VdASPF2 were important multifunction factors in the pathogenicity of V. dahliae and relative to stress adaptation and activated plant immune response.

**IMPORTANCE** Verticillium wilt, caused by the notorious fungal pathogen V. dahliae, is one of the main limiting factors for agricultural production. Metalloproteases played an important role in the pathogenic mechanism of pathogens. Our research found that M35 family metalloproteases VdM35-1 and VdASPF2 played an important role in the development, adaptability, and pathogenicity of V. dahliae, providing a new perspective for further understanding the molecular mechanism of virulence of fungal pathogens.

## INTRODUCTION

Cotton is an important natural fiber crop and plays an essential role in the national economy. As a vascular bundle infection disease, Verticillium wilt caused by Verticillium dahliae seriously affects cotton production and is widely distributed worldwide, known as cotton cancer (1). Symptoms include chlorosis and wilt of leaves or defoliation, vascular bundle browning, and even death, ultimately. The pathogen has strong variability and coevolutes with the host, so its pathogenic mechanism is very complex. The interaction mechanism between the pathogen and host is still unclear, which is a key problem for effective prevention and control of the disease (2, 3). Therefore, in-depth understanding of the molecular pathogenic mechanism of V. dahliae is beneficial to control Verticillium wilt of cotton.

The plant immune response is mainly composed of two layers of defense response in plants (4). The first layer defense system is a disease-resistant response triggered by pattern recognition receptors (PRRs) on the plant cell membrane surface that recognize the conserved pattern molecules of pathogenic microorganisms (microbe-associated molecular patterns [MAMPs] or pathogen-associated molecular patterns [PAMPs]), which is called PTI (PAMP-triggered immunity) (5). The second layer of defense response is that the nucleotide-binding leucine-rich repeat (NB-LRR) type of disease-resistant proteins in plant cells directly or indirectly identify the effectors secreted by pathogenic microorganisms into plant cells, triggering a stronger immune response, which is called effector-triggered immunity (ETI) (6). Plant PTI, also known as basic resistance, is a series of reactions through which plants perceive PAMPs of pathogenic microorganisms through receptor-like kinases (RLKs) or receptor-like proteins (RLPs) on the cell membrane surface, thereby triggering plant cells (7). For example, the effector proteins Vd424Y, VdEG1, and VdXyn4 secreted by V. dahliae were recognized by the core immune receptors on the plasma membrane of plants, which triggered the immune response of plants and upregulated the expression of disease-related genes PRs (8–10). Effector proteins secreted by pathogens inhibit plant immune responses in many ways, including PTI inhibition (11), so that pathogens can infect plants successfully (12–15). In order to resist the pathogenic microorganism, host cells trigger ETI by recognizing effectors (16). ETI often produces large amounts of peroxides (such as reactive oxygen species [ROS]) in infected plant cells and induces hypersensitive response (HR) in plants (16).

Effector proteins play an important role in the process of pathogen infection. Most effector proteins have conserved motifs at the N or C terminus, such as RXLR, CRN, CFEM, RGD, DELD, RYWT, or Y/F/WXC (3, 17, 18). V. dahliae can secrete large amounts of effector proteins into the host. For example, VdNEP, PevD1, or VdCP1 can induce plant cell death and trigger immune response (9, 19, 20). In addition, metalloproteases play important roles in the pathogenic mechanisms of pathogens, which were widely distributed in many pathogens such as V. dahliae and Fusarium oxysporum (21). Among them, the zinc ion-dependent metalloprotease contained conserved HEXXH domain, and two histidine residues were used as the first and second zinc ligands, respectively (22). F. oxysporum metalloprotease FoMep1 and serine protease FoSep1 had a synergistic effect on the chitinase cleavage of tomato, thereby reducing antifungal activity and enhancing virulence of pathogens (23). Most M35 families belong to zinc ion-dependent metalloproteases secreted by pathogens. Avr-Pita of the M35 family of Magnaporthe grisea proved to be an effector protein interacting with Pi-ta in plant cells to trigger host defense response (24). Rhizoctonia cerealis M35 family RcMEP2 and an entomopathogenic fungus Metarhizium anisopliae M35-4 had secretory activity and played a role in host infection as pathogenic factors (25, 26). Previous studies have shown that V. dahliae VdASPF2 secreted protein with M35 family conserved domain. VdASPF2 induced microsclerotia production and melanin accumulation under nutrient stress and low-temperature conditions, resulting in the decrease of pathogenicity of the deletion mutant to cotton (27).

In this study, a metalloprotease VdM35-1 containing the conserved domain of M35 family was identified in V. dahliae, which contained a signal peptide. The conserved motif HEXXH in VdM35-1 was similar to the conserved motif HRXXH of the published zinc ion-dependent metalloprotease VdASPF2 (28). Both could induce cell death in Nicotiana benthamiana leaves and were mainly located on the cell membrane and induced cell death depending on two histidine residues and signal peptides. Cell death induced by VdM35-1 was dependent on BAK1 and SOBIR1, while the cell death induced by VdASPF2 was independent of BAK1 and SOBIR1. In addition, ΔVdM35-1 and ΔVdASPF2 mutants exhibited more sensitivity to potassium chloride (KCl), sodium chloride (NaCl), sorbitol, SDS, Congo red (CR), and high temperatures. These results of different carbon source utilization experiments showed that the utilization capacities of ΔVdM35-1 and ΔVdASPF2 for sucrose, raffinose, and starch were decreased, especially on galactose-containing medium. Disease symptom observations showed that the virulence of deletion mutants to cotton was significantly reduced compared with that of the wild type strains. Collectively, these results showed that metalloproteases VdM35-1 and VdASPF2 positively regulated the virulence of V. dahliae and played an important role in the normal nutritional growth and stress response.

## RESULTS

### Identification of two secreted proteins from V. dahliae that induced plant cell death.

**FIG 1:**
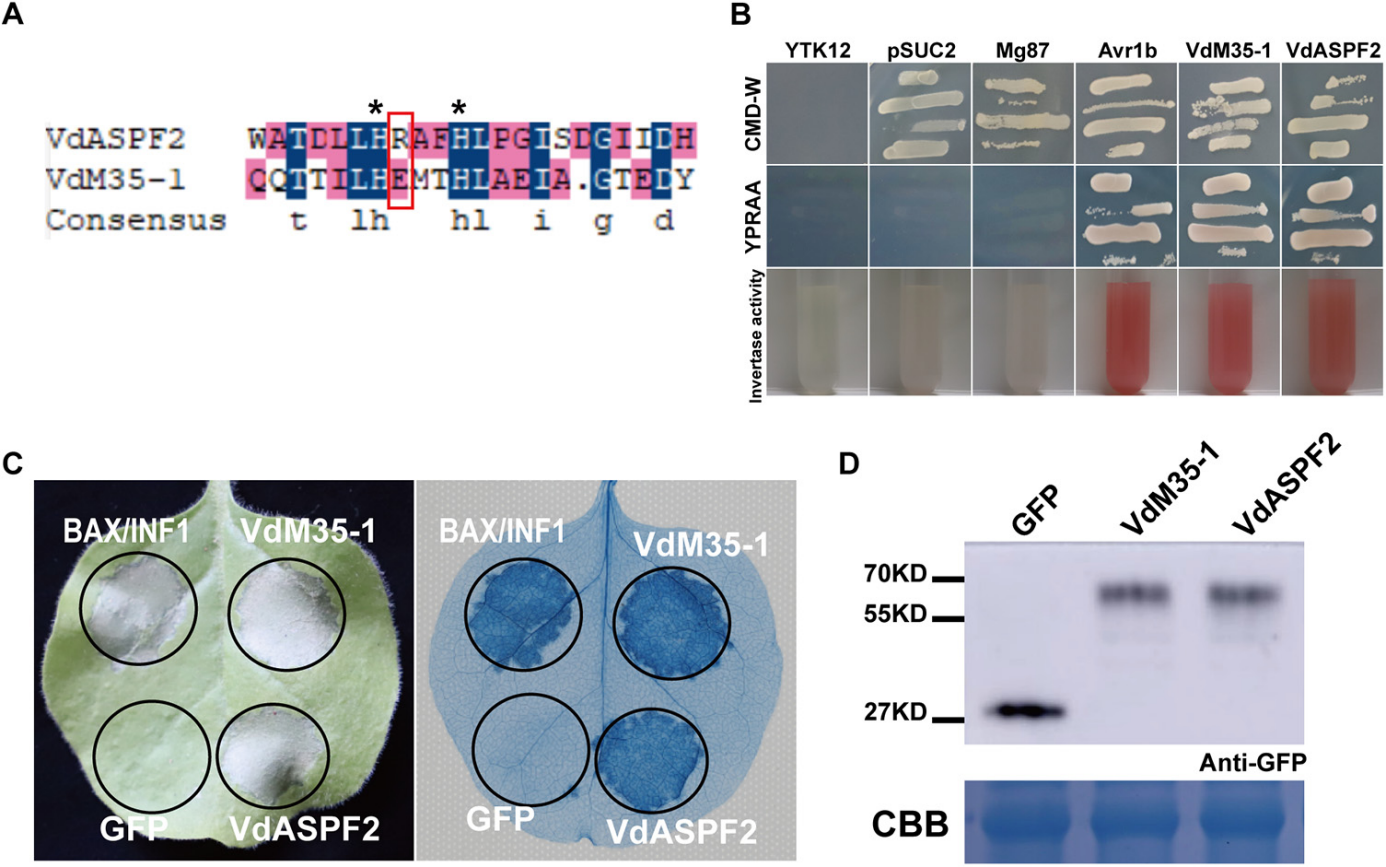
Identification of two secreted proteins from V. dahliae that induced plant cell death. (A) Comparison of amino acid sequences of VdM35-1 and VdASPF2. Residues that are the same are expressed in blue; similar residues are expressed in pink; and histidine residues are marked with asterisks. (B) Signal peptide capture test to verify the signal peptide functions of VdM35-1 and VdASPF2. (C) VdM35-1 and VdASPF2 induced cell death. PVX-GFP, PVX-VdM35-1, PVX-VdASPF2, PVX-BAX, and PVX-INF1 were expressed in 4-week-old N. benthamiana, respectively. Photographs were taken 9 days after Agrobacterium injection. Trypan blue staining was performed to verify cell death. (D) The transient expression of VdM35-1, VdASPF2, and green fluorescent protein were detected by Western blotting. The proteins were stained with Coomassie brilliant blue to determine equal loading.

A metalloprotease (VDAG_00129) was identified in the V. dahliae strain VdLs17 (https://www.ncbi.nlm.nih.gov/genome/832), which had a conserved motif of M35 family and domain HEXXH named VdM35-1. In the previous reports, VdASPF2 (VDAG_04551) containing the conserved domain of M35 family also had a similar conserved motif HRXXH (27, 28) (Fig. 1A). The full-length cDNA of VdM35-1 and VdASPF2 was cloned from the strong pathogenic defoliating strain Vd080. The N-terminal signal peptides of VdM35-1 and VdASPF2 were predicted by SignaIP 5.0, and the yeast signal trap system assay showed that YTK12-pSUC2-VdM35-1SP and VdASPF2SP exhibited the normal growth on CMD-W and YPRAA medium, which suggested that VdM35-1 and VdASPF2 were most likely secreted into the extracellular space during infection (Fig. 1B). Furthermore, the subcellular localization in plants of the two candidate effectors showed that VdM35-1 (full-length amino acid sequence) and VdASPF2 (full-length amino acid sequence) were mainly located on the cell membrane of plants (Fig. S1A and B). Their localization on plant cell membrane were further verified by onion plasmolysis (Fig. S1C). Then, cell death experiments were performed in N. benthamiana leaves. The apoptosis-inducing related proteins (BAX and INF1) and empty vector green fluorescent protein (GFP) were used as positive and negative controls, respectively. The results showed that both VdM35-1 and VdASPF2 could induce cell death (Fig. 1C). Both candidates were confirmed to be expressed in N. benthamiana by Western blotting (Fig. 1D). The electrolyte leakage around the VdM35-1 and VdASPF2 expression regions were significantly increased compared to the GFP expression region. It was found that both VdM35-1 and VdASPF2 could trigger the burst of ROS (Fig. S2). These results suggested that both VdM35-1 and VdASPF2 could induce cell death.

### Cell death induced by VdM35-1 and VdASPF2 depending on two histidine residues and SP.

**FIG 2:**
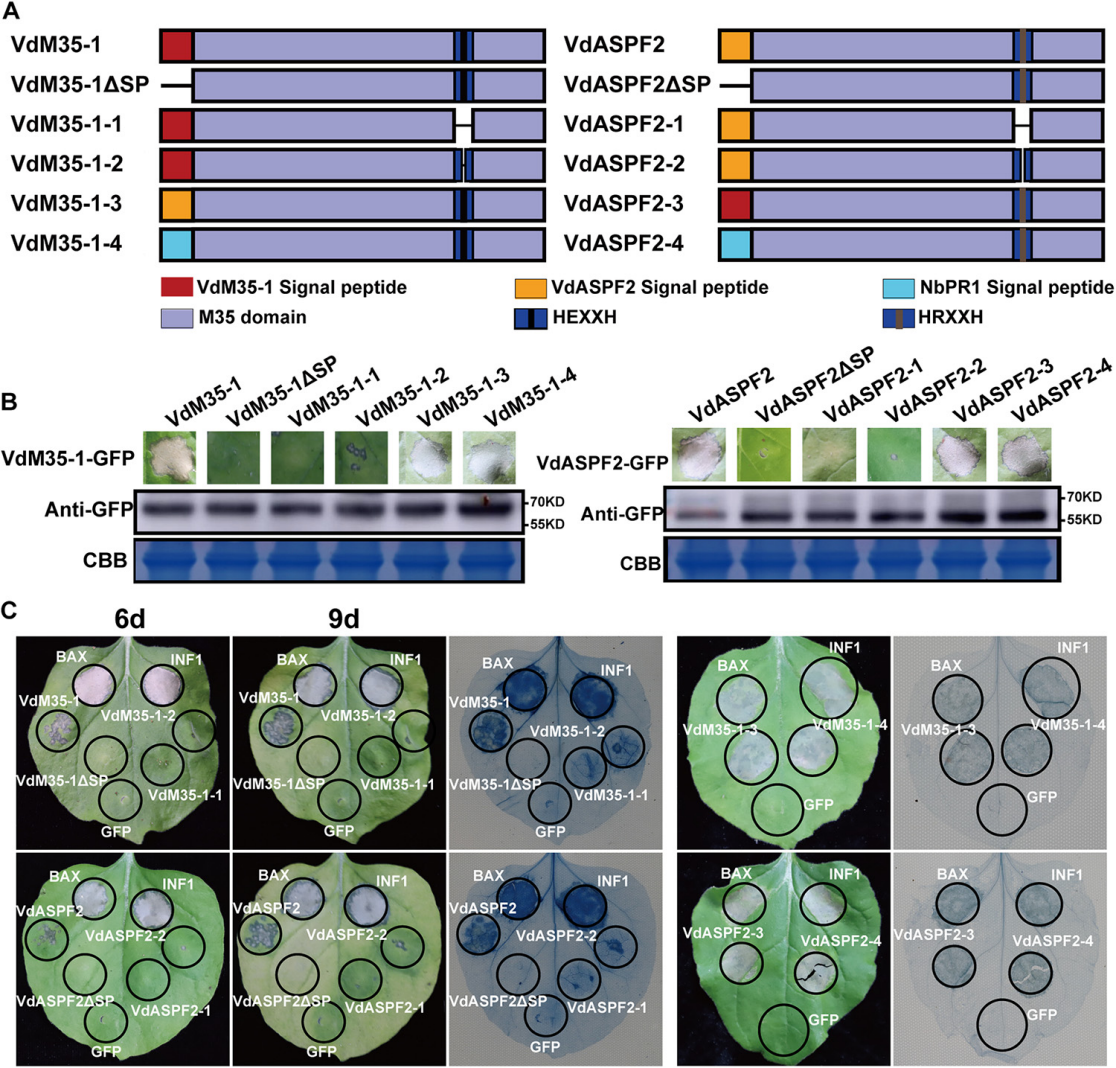
Cell death induced by VdM35-1 and VdASPF2 depending on two histidine residues and the signal peptide (SP). (A) VdM35-1 and VdASPF2 mutant map: VdM35-1 and VdASPF2, candidate effector protein full-length sequence; VdM35-1-ΔSP and VdASPF2-ΔSP mutants lacking signal peptide sequences; VdM35-1-1 and VdASPF2-1, mutants lacking HEXXH and HRXXXH sequences; VdM35-1-2 and VdASPF2-2 mutants lacking E and R sequences; VdM35-1-3 and VdASPF2-3, two protein signal peptide exchange position mutants; and VdM35-1-4 and VdASPF2-4 were mutants fused with NbPR1 signal peptide, a N. benthamiana pathogenic-related protein. (B) Western blot detection of transient expression proteins of VdM35-1, VdASPF2, and all mutants. The proteins were stained with Coomassie brilliant blue to determine equal loading. (C) Transient expression results of VdM35-1, VdM35-1-ΔSP, VdM35-1-1, VdM35-1-2, VdM35-1-3, VdM35-1-4, VdASPF2-ΔSP, VdASPF2-1, VdASPF2-2, VdASPF2-3, VdASPF2-4, positive-control BAX, positive-control INF1, and negative-control green fluorescent protein (GFP) in N. benthamian. Trypan blue staining to verify cell death.

To determine whether VdM35-1- and VdASPF2-induced cell death was dependent on the secretion of VdM35-1 and VdASPF2, the signal peptide (SP) deletion mutants (VdM35-1^17–355^ and VdASPF2^19–297^; here, VdM35-1ΔSP and VdASPF2ΔSP) and four protein signal peptide exchange mutants (VdM35-1-SP^ASPF2^, VdASPF2-SP^M35-1^, VdM35-1-SP^NbPR1^, and VdASPF2-SP^NbPR1^; here, VdM35-1-3, VdASPF2-3, VdM35-1-4, and VdASPF2-4) were constructed (Fig. 2A). All six candidates were confirmed to be expressed in N. benthamiana by Western blotting (Fig. 2B). The results showed that VdM35-1ΔSP and VdASPF2ΔSP did not induce cell death, but the expression of VdM35-1-3, VdASPF2-3, VdM35-1-4, and VdASPF2-4 could all induce cell death (Fig. 2C). For the detection of hydrogen peroxide production and electrolyte leakage in N. benthamiana, all results were consistent with the phenotype (Fig. S2A and B). This suggested that SP was necessary for VdM35-1- and VdASPF2-induced cell death.

VdM35-1 contained the conserved motif HEXXH, while VdASPF2 had the conserved motif HRXXH. To determine whether VdM35-1 and VdASPF2-induced cell death depends on conserved motifs or two histidine residues (H), we constructed mutants with deletions of HEXXH, HRXXH, E site, and R site (VdM35-1^1–306, 312–355^, VdASPF2^1–188, 194–297^, VdM35-1^1–307, 309–355^, and VdASPF2^1–189, 191–297^; here VdM35-1-1, VdASPF2-1, VdM35-1-2, and VdASPF2-2; Fig. 2A). All four candidates were confirmed to be expressed in N. benthamiana by Western blotting (Fig. 2B). The results showed that VdM35-1-1 and VdASPF2-1 did not induce cell death, whereas expression of both VdM35-1-2 and VdASPF2-2 could slightly induce cell death (Fig. 2C). For detection of H_2_O_2_ production and electrolyte leakage in N. benthamiana, all results were consistent with the phenotype (Fig. S2A and B). These results showed that VdM35-1 and VdASPF2 required two histidine residues and SP to induce cell death.

### VdM35-1 and VdASPF2 triggered plant immunity response.

**FIG 3:**
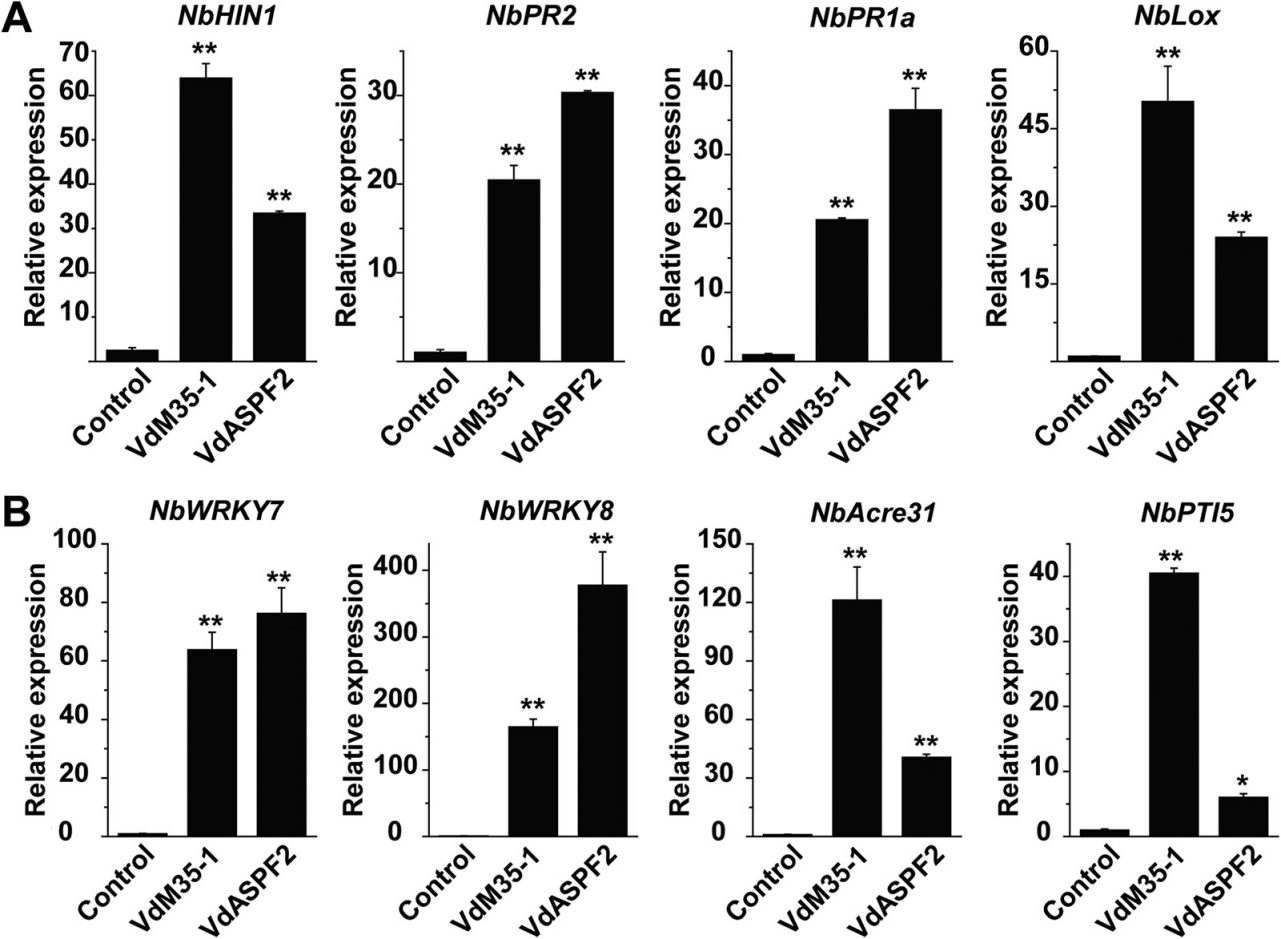
VdM35-1 and VdASPF2 triggered plant immunity response. (A) Relative expression of hypersensitive-response-specific and defense-related marker genes in N. benthamiana infiltrated with Agrobacterium tumefaciens carrying *VdM35-1* and *VdASPF2*. At 3 days postinfiltration (dpi), total RNA was extracted and transcript levels were detected by quantitative reverse transcription-PCR. *NbActin* was used as the internal reference gene. (B) Relative transcript levels of *NbWRKY7*, *NbWRKY8*, *NbAcre31*, and *NbPti5* were analyzed in N. benthamiana infiltrated with A. tumefaciens carrying *VdM35-1* and *VdASPF2*. At 3 days postinfiltration, total RNA was extracted and transcript levels were detected by RT-qPCR. *NbActin* was used as the internal reference gene. The values represent means ± standard deviation of three replicates. *, *P* < 0.05; **, *P* < 0.01.

HR is a type of programmed cell death that is generally considered to be closely related to the plant defense response. To determine whether VdM35-1 and VdASPF2-activated plant immune response, we examined a HR-specific marker gene (*NbHIN1*) in N. benthamiana leaves following VdM35-1 and VdASPF2 infiltration via Agrobacterium. The results showed that *NbHIN1* was significantly activated by VdM35-1 and VdASPF2 at 3 days after infiltration, which indicates that VdM35-1 and VdASPF2 could trigger HR-associated immunity of N. benthamiana. Quantitative reverse transcription-PCR (RT-qPCR) was used to detect the SA-dependent immunity marker genes *NbPR2*, *NbPR1a*, and *NbLOX*. The relative expression of these genes increased significantly (Fig. 3A). Therefore, VdM35-1 and VdASPF2 could induce plant immune response by activating SA-mediated defense pathways.

Because VdM35-1 and VdASPF2 could induce apoptosis and trigger plant immune response, we speculated that VdM35-1 and VdASPF2 may play a role as a PAMP. Therefore, the relative expression of PTI marker genes was measured in this experiment. The results showed that the relative expression levels of PTI marker genes *NbWRKY7*, *NbWRKY8*, *NbACRE31*, and *NbPTI5* were significantly increased after VdM35-1 and VdASPF2 were infected by Agrobacterium (Fig. 3B), respectively, indicating that VdM35-1 and VdASPF2 may be PAMPs, and they participated in plant-induced immune response through a PTI process.

### VdM35-1, rather than VdASPF2, induced cell death in N. benthamiana depending on NbBAK1 and NbSOBIR1.

**FIG 4:**
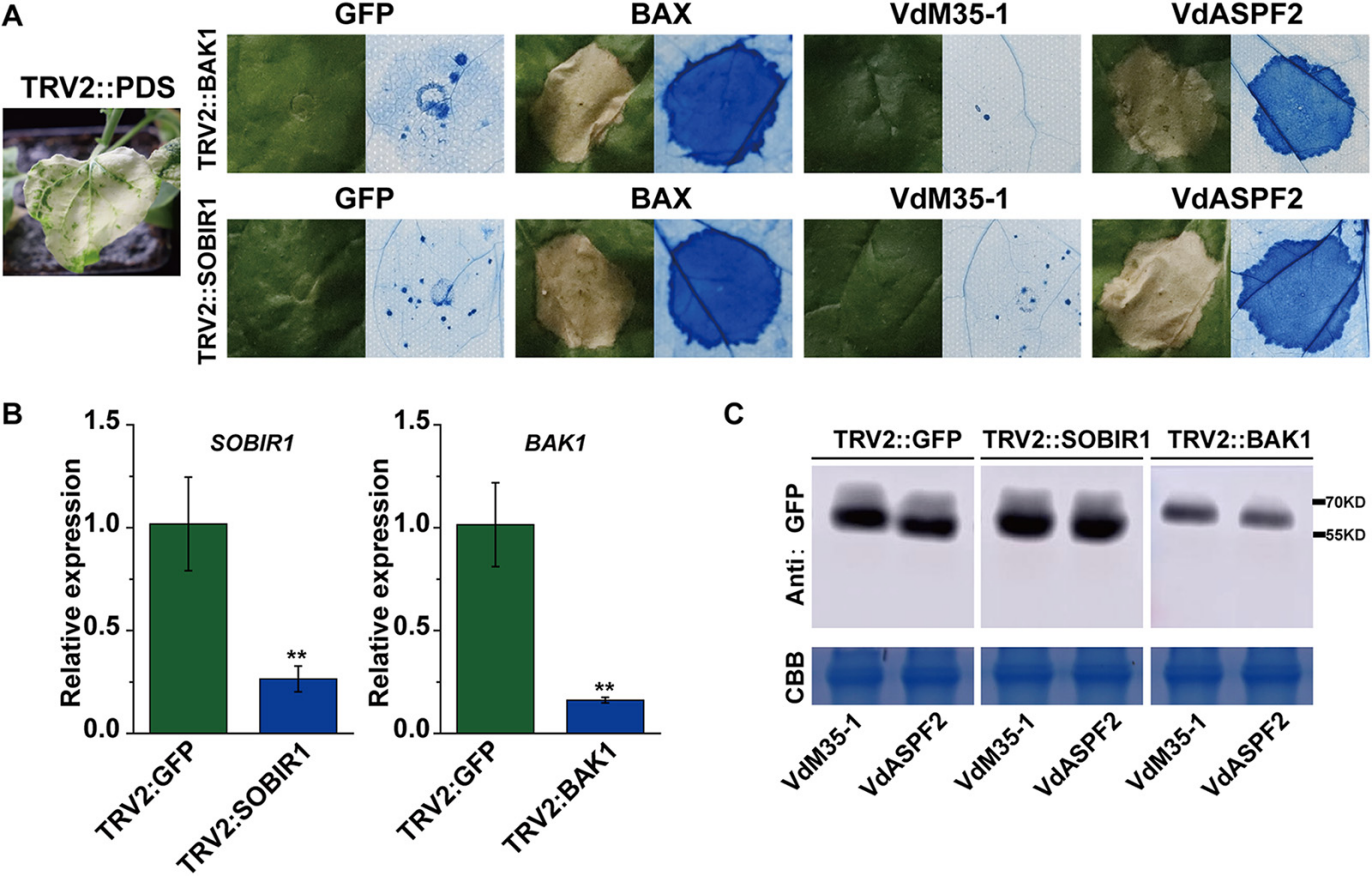
VdM35-1, rather than VdASPF2, induced cell death in N. benthamiana depending on NbBAK1 and NbSOBIR1. (A) Transient expression of VdM35-1 and VdASPF2 in BAK1 and SOBIR1 silenced of N. benthamiana plants. (B) Proteins are stained with Coomassie brilliant blue to determine equal loading. RT-qPCR results of *BAK1* and *SOBIR1* gene silencing efficiency. *, *P* < 0.05; **, *P* < 0.01. (C) Western blot detection of transient expression proteins of VdM35-1 and VdASPF2.

BAK1 and SOBIR1 are membrane receptor-associated kinases involved in immune responses triggered by some PAMPs in plants. In order to verify whether NbBAK1 and NbSOBIR1 mediate VdM35-1 and VdASPF2 induced cell death in N. benthamiana, virus-induced gene silencing (VIGS) was performed to silence *NbBAK1* and *NbSOBIR1* in N. benthamiana leaves, and RT-qPCR was used to confirm the silencing of *NbBAK1* and *NbSOBIR1* at 21 days after VIGS-mediated gene silencing (Fig. 4B). Then, VdM35-1, VdASPF2, and BAX were transiently expressed in N. benthamiana leaves of silenced *NbBAK1* and *NbSOBIR1*, and the expression of VdM35-1 and VdASPF2 was confirmed by Western blotting analysis (Fig. 4C). The results showed that BAX and VdASPF2 retained their ability to induce cell death. In contrast, VdM35-1 no longer induced cell death (Fig. 4A). These results suggested that VdM35-1-induced cell death was dependent on *NbBAK1* and *NbSOBIR1*, whereas VdASPF2-induced cell death was independent of *NbBAK1* and *NbSOBIR1*.

### VdM35-1 and VdASPF2 were essential for normal vegetative growth and conidial production.

**FIG 5:**
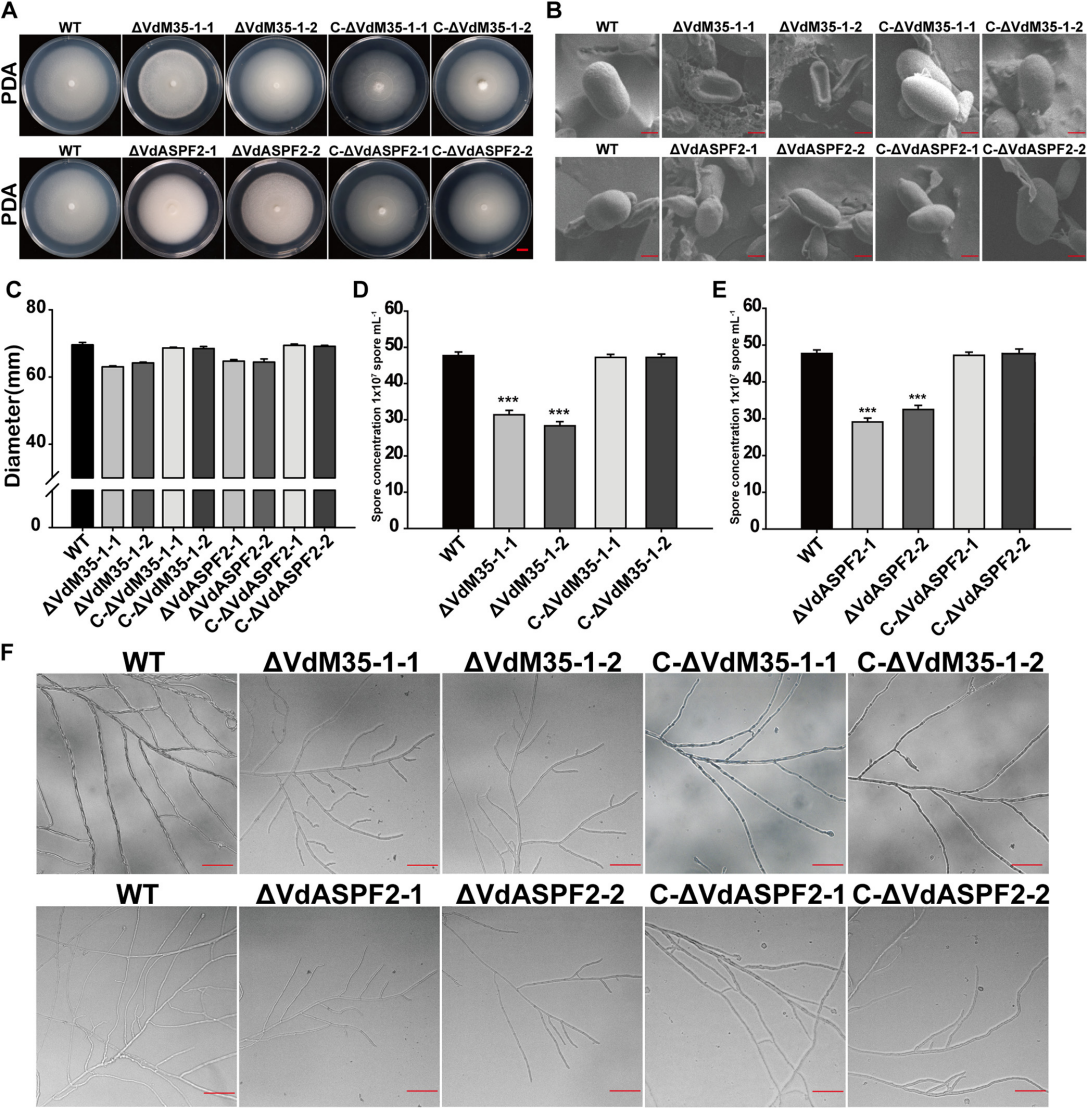
VdM35-1 and VdASPF2 were essential for normal vegetative growth and conidial production. (A) Colony morphology of all strains cultured on PDA plates at 25°C in dark for 14 days. Bar, 1 cm. (B) Morphology of conidia of all strains under scanning electron microscope. Bars, 4 μm. (C) Colony diameter of all strains. (D, E) Sporulation of all strains. The data show averages of three independent repetitions. (F) The mycelium morphology of all strains on PDA plate under fluorescence microscope. Bars, 100 μm. WT, wild type.

In order to study the functions of VdM35-1 and VdASPF2, the gene knockout mutants were constructed by homologous recombination (Fig. S3A). In all transformants, deletion of *VdM35-1* and *VdASPF2* was confirmed by PCR, two mutants of each gene (ΔVdM35-1-1 and ΔVdM35-1-2, ΔVdASPF2-1, and ΔVdASPF2-2) were selected for further investigation (Fig. S3B), and hygromycin (HPH) was verified to be single copy by Southern blotting (Fig. S3D). Complementary strains (C-ΔVdM35-1-1, C-ΔVdM35-1-2, C-ΔVdASPF2-1, and C-ΔVdASPF2-2) were also obtained by homologous recombination (Fig. S3C). The radial growth of ΔVdM35-1 and ΔVdASPF2 strains on potato dextrose agar (PDA) was similar to those of wild type strain (Fig. 5A and C). In addition, the number of conidia produced by two ΔVdM35-1 and two ΔVdASPF2 strains on PDA were lower than the wild type strain (Fig. 5D and E). The observation of spore morphology showed that ΔVdM35-1 led to spore malformation and presented the shape of a concave disk, while the spore morphology of ΔVdASPF2 was similar to the wild type strain (Fig. 5B). The hyphal round branch ends of ΔVdM35-1 and ΔVdASPF2 were shorter than those of the wild type strains (Fig. 5F).

In order to detect the ability of ΔVdM35-1 and ΔVdASPF2 to utilize different carbon sources, the growth rates were monitored on the observation medium with sucrose, galactose, raffinose, and starch as carbon sources. The wild type strain Vd080 grew well in all carbon source tests. The growth rate of knockout mutants on medium containing sucrose and starch were lower than that of wild type strains (Fig. S4). Furthermore, the growth of ΔVdM35-1 and ΔVdASPF2 on galactose-containing medium were significantly limited, with colony diameters of 50 and 47% of wild type strains, respectively (Fig. S4). These results showed that under normal growth conditions, the deletion of VdM35-1 and VdASPF2 not only led to the decrease of conidial yield but also affected mycelial growth and reduced the ability of V. dahliae to utilize different carbon sources.

### VdM35-1 and VdASPF2 participated in stress adaptation.

**FIG 6:**
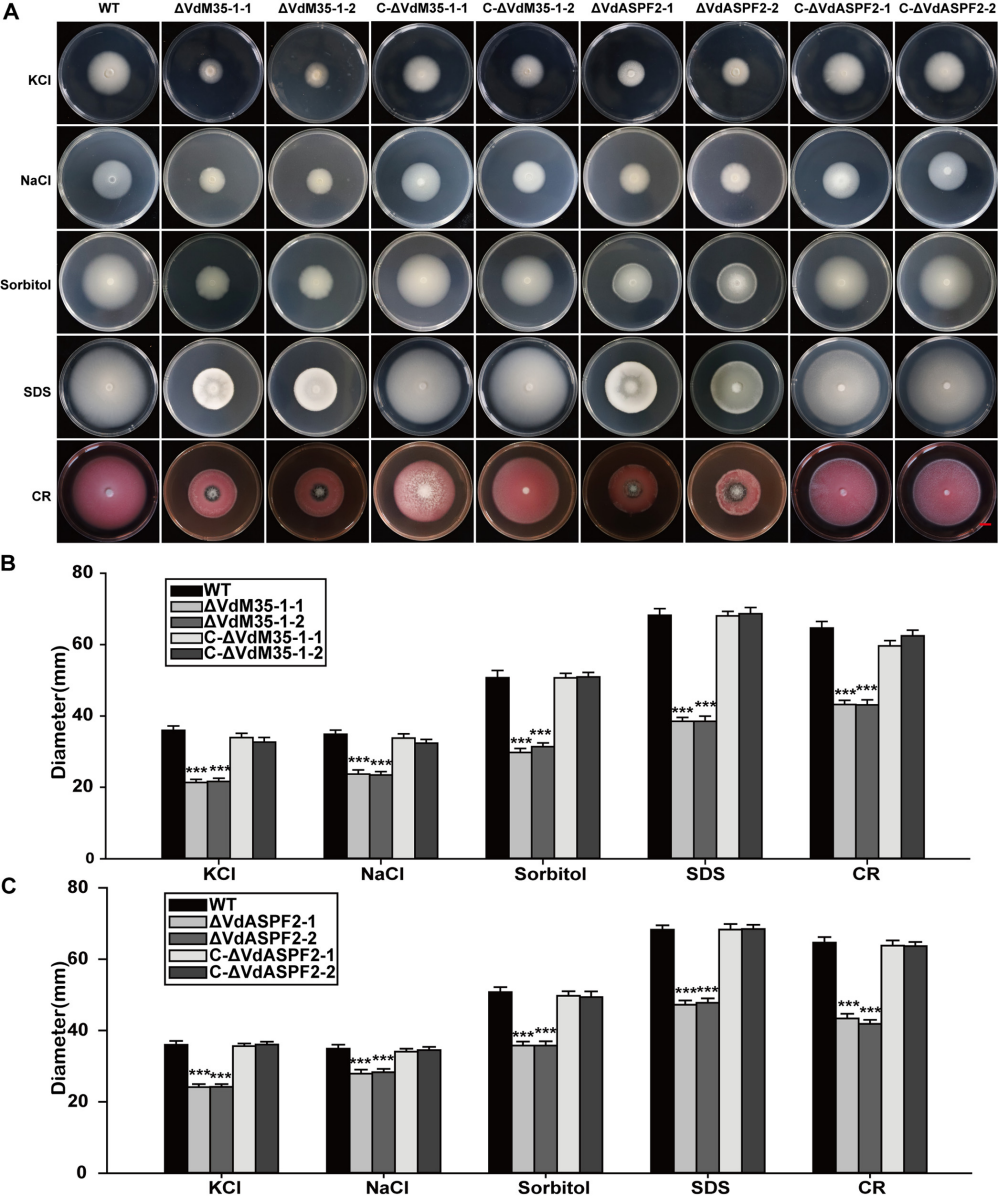
VdM35-1 and VdASPF2 participated in stress adaptation. (A) All strains were cultured on PDA plates supplemented with 1 mol/liter potassium chloride, 1 mol/liter sodium chloride, 1 mol/liter sorbitol, 0.002% SDS, and 0.02% Congo red (CR) at 25°C for 14 days. Bar, 1 cm. (B, C) Colony diameters of all strains. The values represent means ± standard deviation of three replicates. The asterisks represent statistical differences performed by a *t* test in comparison with the wild type strains: *, *P* < 0.05; **, *P* < 0.01; ***, *P* < 0.001.

In order to detect the response of ΔVdM35-1 and ΔVdASPF2 to different stresses, the growth rates were monitored on the PDA medium containing KCl, NaCl, sorbitol, SDS, and CR, respectively. The colony diameters of ΔVdM35-1 and ΔVdASPF2 were smaller and more sensitive to stress (Fig. 6A to C). The growth of wild type strain Vd080 on PDA plates containing SDS and CR were similar to that on normal PDA plates, but the growth of ΔVdM35-1 and ΔVdASPF2 were inhibited, and the colony diameters were 56, 67, 71, and 66% that of wild type strains, respectively (Fig. 4A and 5A). ΔVdM35-1 was more sensitive to sorbitol and SDS stress than ΔVdASPF2. After the spores of each strain were treated at 45 and 4°C for an hour, spore germination was observed at different time intervals. After high-temperature treatment, spores of wild type strain Vd080 germinated and began to form hyphae at 6 h, while spores of ΔVdM35-1 and ΔVdASPF2 germinated and began to form hyphae at 12 h (Fig. S5A). Compared with the wild type strain Vd080, it was found that high-temperature treatment delayed the spore germination of ΔVdM35-1 and ΔVdASPF2. The time of spore germination after low-temperature treatment was the same as wild type strain Vd080 (Fig. S5B). After 3 min of UV irradiation, the spore germination of each strain was observed at different time points. It was found that, like the wild type strain Vd080, the spores of ΔVdM35-1 and ΔVdASPF2 germinated at 6 h and began to form hyphae. At 12 h, hyphae formed branches near the spores (Fig. S5B). These results showed that under stress conditions, the deletion of VdM35-1 and VdASPF2 would lead to more sensitivity of V. dahliae to stress.

### VdM35-1 and VdASPF2 were not involved in mycelium penetration.

**FIG 7:**
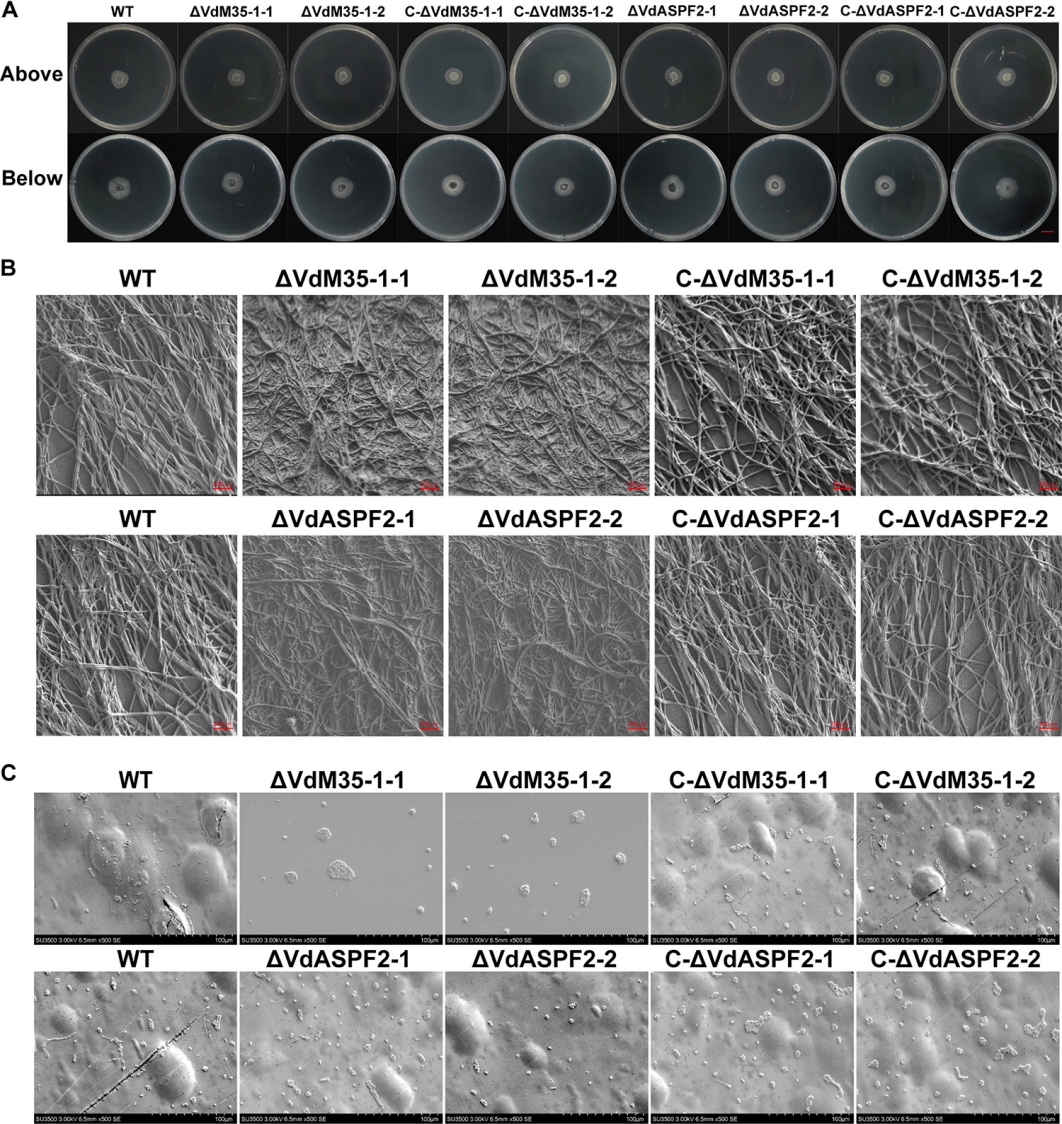
VdM35-1 and VdASPF2 did not involve in mycelium penetration. (A) All the strains were grown on cellophane for 3 days (top) and removal of the cellophane for 3 days (bottom). Bar, 1 cm. (B) Observation of mycelial development on cellophane at 3 days. (C) Observation of conidia on the back of cellophane at 3 days.

In order to detect the penetration ability of ΔVdM35-1 and ΔVdASPF2 on cellophane, equal amounts of conidia of each strain were coated on cellophane laid on PDA medium for 3 days. The results showed that all strains could grow normally on cellophane (Fig. 7A). Three days after removing the cellophane, ΔVdM35-1 and ΔVdASPF2 were observed to grow. Observation of mycelium growth on cellophane showed that the mycelium growth of the knockout mutants was sparse and chaotic, while the mycelium of wild type strain and complemented strain was uniform and normal (Fig. 7B). Conidia of all strains were observed on the back of the cellophane (Fig. 7C). These results indicated that VdM35-1 and VdASPF2 did not affect the penetration of mycelium.

### VdM35-1 and VdASPF2 positively regulated pathogenicity.

**FIG 8:**
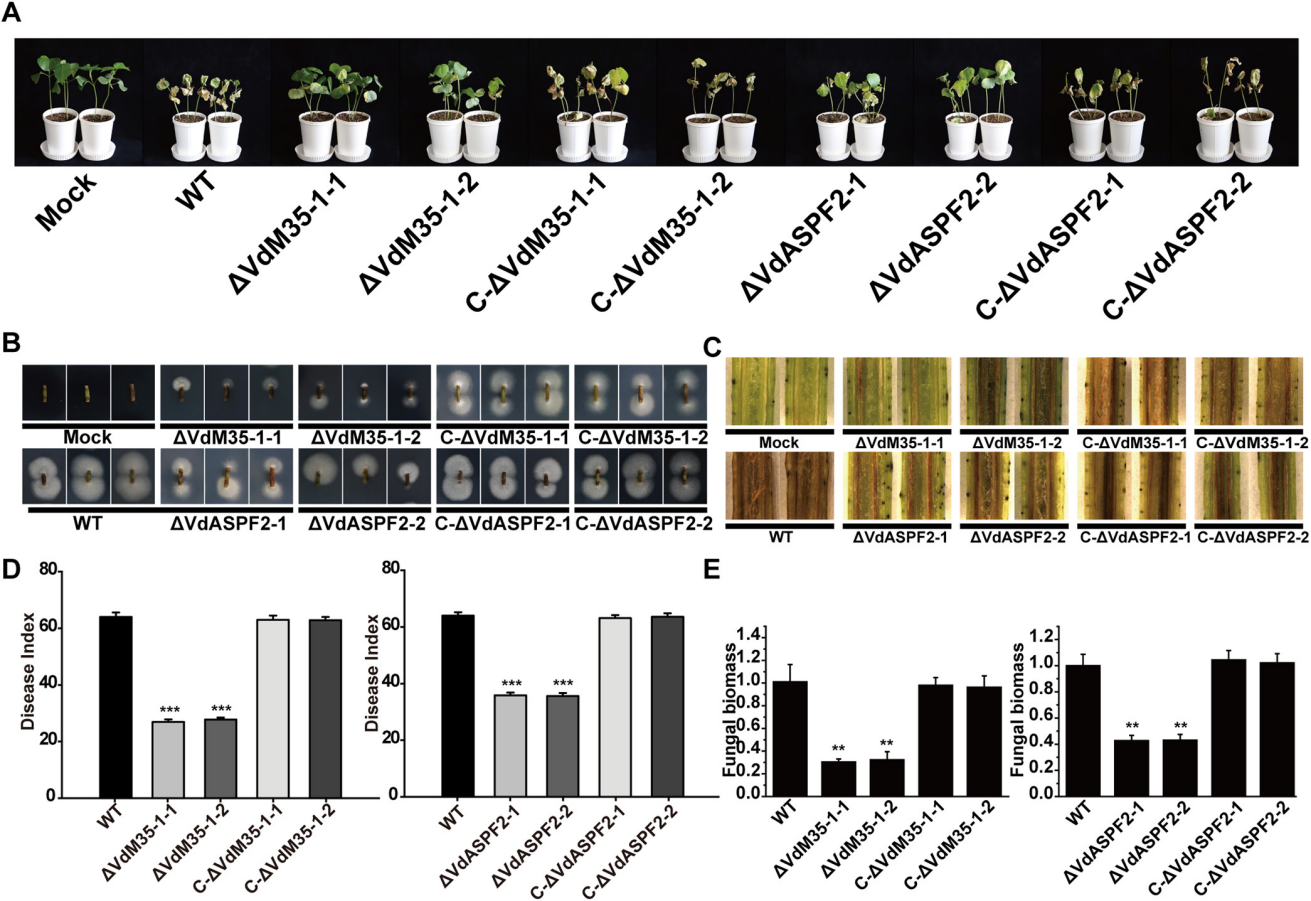
VdM35-1 and VdASPF2 positively regulated pathogenicity. (A) Incidence of cotton plants infected by all strains on 21 days. (B) The stems of diseased cotton at 21 days were cultured on PDA medium for 5 days to isolate V. dahliae again. (C) Vascular discoloration of cotton stem tissue. (D) Investigation on disease index of all strains infected cotton plants at 21 days. The asterisks represent statistical differences performed by a *t* test in comparison with the wild type strains: *, *P* < 0.05; **, *P* < 0.01; ***, *P* < 0.001. (E) Quantitative analysis of fungal DNA in cotton stems at 21 days. *Vdβt* was used as the detection gene, and *Act* gene of upland cotton was used as the endogenous control gene.

During the infection of cotton by V. dahliae, the expression levels of *VdM35-1* and *VdASPF2* in the early stage were significantly increased (Fig. S6). In order to detect the roles of VdM35-1 and VdASPF2 in virulence, the wild type, knockout mutants, and complemented mutants were used for pathogenicity tests on susceptible upland cotton Jimian11. The results showed that the seedlings of cotton wilted, yellowed, and even dropped leaves after inoculation with conidia suspension of wild type strain and complementation mutant strain. After inoculation with ΔVdM35-1 or ΔVdASPF2 conidial suspension, cotton seedlings showed slight wilting and yellowing (Fig. 8A). At 21 days postinoculation, the disease indexes (DIs) of cotton plants infected by deletion mutants (ΔVdM35-1-1, ΔVdM35-1-2, ΔVdASPF2-1, and ΔVdASPF2-2) were 27.05 ± 0.90, 27.90 ± 0.70, 36.04 ± 0.96, and 35.76 ± 1.11, respectively, while that of cotton plants infected by wild type, C-ΔVdM35-1-1, C-ΔVdM35-1-2, C-ΔVdASPF2-1, and C-ΔVdASPF2-2 were 64.22 ± 1.56, 63.18 ± 1.51, 63.06 ± 1.12, 63.33 ± 1.05, and 63.80 ± 1.25, respectively (Fig. 8D). The knockout mutants had slight more browning of the vascular bundle than wild type and complemented mutants, but the vascular bundle browning degree of plants by ΔVdASPF2 infection was more serious than that of ΔVdM35-1 infection (Fig. 8C). The fungal recovery test verified that each strain caused plant disease after infection (Fig. 8B). According to the fungal setting values, the fungal biomass of knockout mutants were lower than those of wild type and complemented mutants. In addition, compared with wild type Vd080, the colonization of ΔVdM35-1 and ΔVdASPF2 strains in cotton roots was also reduced (Fig. 8E). These results suggest that VdM35-1 and VdASPF2 positively regulate the virulence of V. dahliae and indicate that VdM35-1 played a more important role than VdASPF2 in the virulence of V. dahliae.

## DISCUSSION

V. dahliae is a vascular fungal pathogen, which can destroy the vascular bundle of host cells and cause damage to plants (2, 29). Relatively few secretory effector proteins were found to play a role in virulence. Metalloproteinases are considered important virulence factors in fungal pathogens (30, 31), which are widely distributed as in V. dahliae, F. oxysporum, and other pathogens (21). Previous studies found that M35 family metalloproteinases had a conserved motif of HEXXH, which were zinc-dependent metalloproteinases and played an important role in the interaction between pathogens and hosts (32). However, the function of M35 family metalloproteases in V. dahliae were still unclear. In this study, a metalloprotease VdM35-1 containing the conserved motif of HEXXH and one metalloprotease VdASPF2/VDAL containing the conserved motif of HRXXH were found (Fig. 1A). Previous studies showed that VdASPF2 contained the conserved motif of HRXXH (27) and had the binding activity of zinc ions (28). Both VdM35-1 and VdASPF2 had the ability to induce the plant immunity of cell death by targeting plant explants.

Studies have shown that many key interactions between plants and pathogens play a role in the space of apoptosis (33, 34). Like PAMP, Vd424Y of V. dahliae and FoEG1 of F. oxysporum could induce cell death, and their signal peptides were required. When triggering plant defense response, the related genes were upregulated (8, 35). FocM35-1 identified from F. oxysporum can trigger the accumulation of ROS when inducing cell death (32). This study found that VdM35-1 and VdASPF2 had similar functions and lost the ability to induce cell death after the signal peptide was truncated. In addition, subcellular localization showed that VdM35-1 and VdASPF2 were located in plant cell membrane (Fig. S1). These results showed that the extracellular space was very important for the functions of VdM35-1 and VdASPF2. Previous studies have shown that membrane metalloproteinase plays a role in many signaling pathways (36). VdM35-1 and VdASPF2 played roles in cell membrane through plastids and could trigger plant defense responses, including ROS accumulation. (Fig. S2). VdM35-1 and VdASPF2 significantly activated the upregulation of HR (*NbHIN1*) (37), SA (*NbPR2*, *NbPR1a*, and *NbLOX*) (35, 38), and PTI (*NbWRKY7*, *NbWRKY8*, *NbACRE31*, and *NbPTI5*) (8, 35, 38–40) related marker genes, respectively. (Fig. 3). Previous studies have found that M35 and M36 family metalloproteases contain conserved motif HEXXH, which is a zinc-dependent metalloprotease, and it has been confirmed that they contain zinc-binding activity of VDAL/VdASPF2 from V. dahliae (28, 41). Further, the truncated conserved motifs lost the ability to induce cell death (Fig. 2). These results showed that VdM35-1 and VdASPF2 were death-inducing proteins through exosomes to cell membrane.

The functions of VdM35-1 and VdASPF2 mainly depended on the membrane localization of host cells. Previous studies have shown that fungal effectors trigger plant defense responses through the perception of cell membrane immune receptors BAK1 and SOBIR1 (33, 42). For example, the secretion proteins VdEG1, VdEG3, and Vd424Y of V. dahliae induced cell death in N. benthamiana through BAK1/SOBIR1-mediated signaling pathways (8, 9). However, VdRTX1-mediated plant cell death was independent of BAK1 and SOBIR1 (43). Silencing BAK1 or SOBIR1 genes in this formula showed that VdM35-1-induced plant cell death depended on BAK1 and SOBIR1, while VdASPF2-induced plant cell death was independent of BAK1 and SOBIR1. These results suggested that VdM35-1 may be an ectoplast effector, while VdASPF2 is not (Fig. 4). Although they both had M35 family metalloproteinases domains, they induced plant immunity in different ways.

Mycelium and conidia play important roles in the growth cycle of V. dahliae (44). Normal vegetative growth of V. dahliae is regulated by many genes. Previous studies have shown that the absence of *VdSOD1* and *VdSOD3* in V. dahliae does not affect sporulation, spore morphology, and normal vegetative growth of mycelium (45, 46). Conidia of the ΔVdOGDH mutants were atypically rounded or spherical, and hyphae were irregularly branched and lacked typical whorled branches (47). In this study, it was found the sporulation decreased, and the mycelial branch growth became shorter in VdM35-1 and VdASPF2 deletion mutants (Fig. 5D and E and F). Especially, spore morphology of the ΔVdM35-1 was changed (Fig. 5B). Previous studies have found that deletion of *VdHP1* could also lead to changes in spore morphology and improve the utilization of different carbon sources to maintain the normal vegetative growth (48). However, the ability of V. dahliae to utilize different carbon sources decreased to various degrees when VdM35-1 and VdASPF2 were deleted (Fig. S4). Interestingly, VdM35-1 and VdASPF2 did not affect the ability of V. dahliae to penetrate cellophane (Fig. 7). These results showed that VdM35-1 and VdASPF2 were important factors involving in the vegetative growth of V. dahliae.

Previous studies had shown that VdHog1, VdPbs2, and VdSsk2 were highly responsive to osmotic stress (44, 49, 50). VdUGP and VdSkn7 showed high sensitivity to SDS and CR stress. VdHP1 could enhance the resistance of V. dahliae to UV and high temperature and played an important role in the pathogenicity of pathogens (48). In this study, VdM35-1 and VdASPF2 were found to be involved in regulating the responses of V. dahliae to osmotic stress NaCl, KCl, and sorbitol. ΔVdM35-1 and ΔVdASPF2 were sensitive to osmotic stress, SDS, and CR stress, with growth retardation, thereby reducing its pathogenicity (Fig. 6), which indicated that there were defects in the cell walls of ΔVdM35-1 and ΔVdASPF2, leading to the decrease of its pathogenicity. Collectively, these results showed that VdM35-1 and VdASPF2 may be involved in various stresses of V. dahliae to the environment, helping pathogenic fungi survive in nature.

The metalloprotease is also involved in the regulation of pathogenicity. Previous studies have shown that FocM35_1, RcMEP2-RcMEP5, and MrM35-4 played important roles in pathogenicity (25, 26, 32). After gene deletion, the pathogenicity of deletion mutants was significantly decreased compared to the wild type. In this study, it was found that after inoculation of cotton with conidial suspensions of ΔVdM35-1 and ΔVdASPF2 mutants, the incidence and severity of cotton verticillium wilt were significantly reduced compared to wild type and complemented mutants. By investigating the disease index, measuring fungal biomass, and observing vascular browning, these results showed that the pathogenicity of the knockout mutants were significantly decreased, but the pathogenicity of ΔVdASPF2 was stronger than ΔVdM35-1 (Fig. 8). It is concluded that VdM35-1 and VdASPF2 were positive regulators of virulence and related to the pathogenicity of V. dahliae.

In summary, this study demonstrated that VdM35-1 and VdASPF2 could effectively activate the immune response of plants and regulate the pathogenicity of V. dahliae by participating in vegetative growth, conidia production, and mycelial growth, which suggest that metalloproteinases play an important role in the development, adaptability, and pathogenicity of V. dahliae, and provided a new perspective for further understanding the molecular pathogenicity mechanism of fungal pathogens. Further research is necessary to elucidate the VdM35-1 and VdASPF2 signaling mechanism in V. dahliae and to search for interacting proteins in cotton.

## MATERIALS AND METHODS

### Strains and plants.

The wild type V. dahliae strain Vd080 is a strong pathogenic defoliating strain and is cultured in liquid Czapek Dox medium or on potato dextrose agar medium (48). Upland cotton (Gossypium hirsutum) cultivar Jimian11 was highly susceptive to V. dahliae and was grown in a 28°C temperature-controlled glasshouse (14-h light/10-h dark). N. benthamiana was grown in the growth chamber at 25°C.

### Gene cloning and bioinformatics analysis.

The full lengths of coding sequences of *VdM35-1* (VDAG_00129) and *VdASPF2* (VDAG_04551) were amplified from cDNA of V. dahliae Vd080 using the specific primers (Table S1). Prediction of functional conservative domains of VdM35-1 and VdASPF2 was performed using CDD Tool (https://www.ncbi.nlm.nih.gov/Structure/cdd/wrpsb.cgi).

### Yeast signal sequence trap system.

The yeast signal peptide screening was used to verify the secretory function of effector protein signal peptides (51). The yeast strain chosen for the experiment was YTK12 (52); it lacked the sucrose convertase gene and cannot grow on a medium with sucrose as the only carbon source. The predicted signal peptide was inserted into the vector pSUC2. Transfer of recombinant plasmid to yeast strain YTK12 screened positive clones in CMD-W medium (deletion of tryptophan). Positive clones grown on CMD-W medium were transferred to YPRAA machine (2% raffinose) and incubated upside down at 28°C. The function of signal peptide was screened and verified by YPRAA medium and TTC coloration test. Further, the signal peptide function was validated by chromogenic reaction. The primers used in this assay are listed in Table S1.

### Agrobacterium-mediated transient transformation of N. benthamiana.

Constructed pGR107-VdM35-1, pGR107-VdM35-1-ΔSP, pGR107-VdM35-1-ΔHEXXH, pGR107-VdM35-1-ΔE, pGR107-VdASPF2, pGR107-VdASPF2-ΔSP, pGR107-VdASPF2-ΔHRXXH, pGR107-VdASPF2-ΔR, pGR107-VdM35-1-SP^ASPF2^, pGR107-VdASPF2-SP^M35-1^, pGR107-VdM35-1-SP^NbPR1^, and pGR107-VdASPF2-SP^NbPR1^ were transferred into Agrobacterium tumefaciens receptor cells GV3101 (53, 54). After 2 days of inverted culture, positive clones were picked for seed to 5 mL with relevant resistance in LB liquid medium, shaken at 28°C and 200 r/min for 2 to 3 days until the bacterial solution was cloudy, and then washed three times with 5 mL of Agrobacterium infection solution (55). The 6- to 8-week N. benthamiana plants were inoculated with Agrobacterium (optical density at 600 nm [OD_600_] = 0.8) on the second to fourth leaves. Cell death was observed within 5 to 7 days, and the leaves were photographed 7 to 10 days after inoculation. Protein expression was verified by Western blotting using total protein extracted from leaves. Determination of the degree of cell death by trypan blue staining (56, 57). The primers used in this assay are listed in Table S1. Three independent biological and technical repeats were performed.

### VIGS in N. benthamiana.

In TRV-mediated gene silencing assays, the plasmid constructs pTRV1, pTRV2::NbBAK1, pTRV2::NbSOBIR1, pTRV2, and pTRV2::PDS were introduced into A. tumefaciens GV3101 (53, 54). The two primary leaves of four-leaf-stage N. benthamiana seedlings were injected with a mixture (1:1 ratio) of A. tumefaciens culture (OD_600_ = 1.8) containing pTRV1 and pTRV2::genes. The pTRV2::PDS was used as a positive control, while the pTRV2 served as a control (58, 59). The RNA extracted from leaves was used to validate the efficiency of gene silencing by RT-qPCR. The primers used in this assay are listed in Table S1.

### Subcellular localization analysis.

The recombinant plasmids 35S::VdM35-1 and 35S::VdASPF2 were transformed into A. tumefaciens GV3101, respectively. The resulting strains were expressed in 4-week-old leaves of N. benthamiana using the previously described method (53, 54). After 48 h of Agrobacterium infiltration, green fluorescence of enhanced GFP (eGFP) was detected by 488-nm confocal microscopy. Using biolistic technology, high-pressure gas was used to accelerate the gold powder particles wrapped with recombinant plasmids directly into the onion skin. After 48 h, green fluorescence of eGFP was detected by confocal microscopy. The protoplasts of Arabidopsis thaliana were extracted, and the recombinant plasmids were transformed using PEG-mediated method. After 16 h of treatment under low light, the fluorescence was observed with a laser confocal microscope. The primers used in this assay are listed in Table S1.

### Target gene knockout and complementation.

Knockout and complemented mutants were obtained by the Agrobacterium-mediated method (60, 61). The upstream fragment (Up) ~1-kb and downstream fragment (Down) ~1-kb sequences of *VdM35-1* and *VdASPF2* gene were selected. The upstream and downstream fragments corresponding to each gene were amplified from Vd080 genomic DNA. The hygromycin (Hyg) fragment was amplified in vector B303. These fragments were fused to linearized B303 using recombinant enzymes (ClonExpress Ultra one step cloning kit; Vazyme, Nanjing, China) to construct the vector Up-Hyg-Down-B303-Hyg according to the manufacturer’s instructions. The constructed recombinant plasmid was transformed into Agrobacterium GAL1 and then transformed into V. dahliae. The positive colonies were screened on PDA containing hygromycin and confirmed by PCR with the primers Hyg-F/R. The copy of Hyg was confirmed by Southern blotting. The recombinant plasmid Up-VdM35-1/VdASPF2-Down-pCAMBIA1302 were obtained by the same method. The recombinant vector was transformed into A. tumefaciens and then cotransformed into the knockout strain to screen the positive transformants of the complemented mutants. The primers used in this assay are listed in Table S1.

### Phenotypic analysis of V. dahliae mutants.

The wild type, knockout, and complemented mutant strains of V. dahliae were inoculated in the liquid Czapek Dox medium and cultured at 25°C for 5 days, and the conidial concentrations of each strain were adjusted to be the same. 5 μL conidia were taken and inoculated on a uniform and fresh PDA plate. The plate was placed in a 25°C incubator for inverted culture for 14 days, and the colony diameters were measured and photographed. Each strain was set at least three replicates (48).

In 5-mm colonies, the wild type, knockout, and complemented mutant strains of V. dahliae were obtained on the plate and inoculated on PDA plates. After 3 days of incubation at 25°C, the sterilized coverslips were obliquely inserted into the medium at the edge of the colony at 45°C for 24 h, and then the coverslips were taken out to observe the mycelial morphology of different strains under a stereo microscope (35).

The wild type, knockout, and complemented mutants strains of V. dahliae were inoculated on PDA plates and cultured at 25°C for 7 days. Fungal colonies with diameters of 5 mm were obtained on the plates with a sterile puncher. The strains were inoculated in a liquid Czapek Dox Medium. One fungal cake was inoculated in each bottle and cultured at 25°C for 3 days at 180 r/min. The filtrate was filtered and collected with a sterile gauze. Then, the concentration of conidia was counted under a microscope with a blood cell count plate. Each strain was set with at least three replicates (35). The conidial solution was taken to prepare frozen sections, and the spore morphology was observed by scanning electron microscopy for photographing.

### Carbon source utilization assays.

In order to analyze the utilization of carbon sources of wild type, knockout, and complemented mutants, galactose (10 g/liter), sucrose (30 g/liter), raffinose (10 g/liter), and starch (17 g/liter) were added to Czapek Dox medium with sucrose deficiency, respectively. The experiment was repeated three times.

### Growth of mutants under stress treatments.

In order to detect the sensitivity of wild type, knockout, and complemented mutants strains to cell wall inhibitors and osmotic stress, all strains were cultured in PDA medium containing 1 M NaCl, 1 M KCl, 1 M sorbitol, 0.02% CR, and 0.002% SDS (44, 62, 63). The diameter was measured after 14 days of incubation at 25°C. Each strain was set at least three replicates.

In order to determine the thermal stability and UV treatment, the conidia (1 × 10^7^ CFU/mL) of all strains were collected from Czapek liquid medium for 5 days. 1 mL suspension was treated at 45 or 4°C with UV light for 1 h, 1 h, or 3 min (48), respectively. Then, the suspension was cultured on PDA at 25°C for 0, 6, and 12 h. The germination was observed under microscope. Each strain was set with at least three replicates.

### RT-qPCR analysis.

Total RNA was isolated using polysaccharide polyphenol plant RNA extraction kit (RC411-C1), quantified, and used as a template for reverse transcription with the HiScript II QRT SuperMix for qPCR (+gDNA wiper R223-01) (Vazyme). The RT-qPCR assays were performed using the ChamQ universal SYBR qPCR Master Mix (Q711-02) (64). The NbActin gene was used as internal control. Three independent biological and technical repeats were performed.

### Infection assays.

Using published methods, 4-week-old Jimian11 plants were inoculated with 10 mL of conidia suspension (65). Infected plants were classified into five grades (0, 1, 2, 3, and 4) based on symptoms of cotyledons and new leaves (19, 66, 67). The disease index was calculated according to a previously described protocol (65).

### Mycelial penetration assays.

The conidia were coated on cellophane to observe the difference of penetration ability of different strains. The morphology of mycelium on glass paper was observed by scanning electron microscope (48, 68). The experiment was repeated three times.

### Protein extraction and Western blotting.

The protein was extracted from leaves of 4-week-old N. benthamiana after Agrobacterium infestation. After 36 h of infestation, equal-weight leaves were ground in liquid nitrogen and added with the corresponding volume of radioimmunoprecipitation assay (RIPA) lysis buffer (Beyotime P0013C). The mixture was lysed on ice for 15 min, then centrifuged at 13,000 × *g* for 10 min at 4°C, transferred to a new tube, and hyperinactivated by adding protein buffer to the collected supernatant solution for 5 min. Protein analysis by SDS-PAGE and electroblotting onto polyvinylidene difluoride (PVDF) membranes (69, 70).

### Southern blotting.

The genomic DNA of the wild type, *ΔVdM35-1-1*, *ΔVdM35-1-2*, *ΔVdASPF2-1*, and *ΔVdASPF2-2* were digested by HindIII, then separated by 0.8% agarose gel electrophoresis, and transferred to the Hybond N + nylon membrane. The cDNA of Hyg was labeled with digoxin as a probe for hybridization. The specific steps were carried out according to the DIG high prime DNA labeling and detection starter kit I instructions (Roche) using the transfer membrane method with reference to Southern blotting (48, 71).
