# Supplementary material for: Two Metalloproteases VdM35-1 and VdASPF2 from Verticillium dahliae Are Required for Fungal Pathogenicity, Stress Adaptation, and Activating Immune Response of Host
Source: Microbiol Spectr. 2022 Oct 12;10(6):e02477-22. doi: 10.1128/spectrum.02477-22 (PMC9769895; doi:10.1128/spectrum.02477-22)
Supplement: Supplemental file 1 — Fig. S1 to S6 and Table S1. Download spectrum.02477-22-s0001.pdf, PDF file, 1.1 MB [file spectrum.02477-22-s0001.pdf]

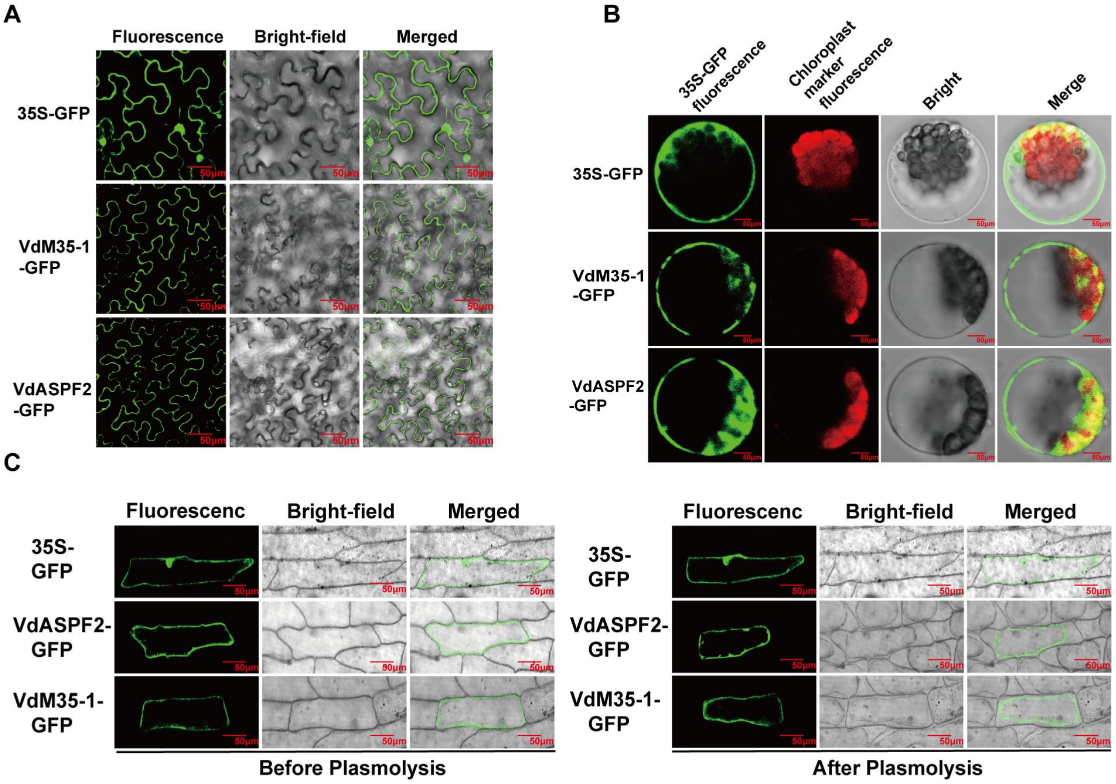

Figure S1: VdM35-1 and VdASPF2 are mainly located in the cell membrane. (A) Subcellular localization of VdM35-1-GFP and VdASPF2-GFP transiently expressed by *Agrobacterium* in *N. benthamian*. After 48h of injection, the fluorescence was detected by confocal microscope. (B) Subcellular localization of VdM35-1-GFP and VdASPF2-GFP recombinant plasmids in Arabidopsis protoplast expression. After 16 hours of expression, fluorescence was detected by confocal microscopy. (C) Using biological technology, tungsten powder particles wrapped by recombinant plasmids of VdM35-1-GFP and VdASPF2-GFP were directly introduced into onion skin by high pressure gas. After 24 hours of expression, fluorescence was detected by confocal microscopy.

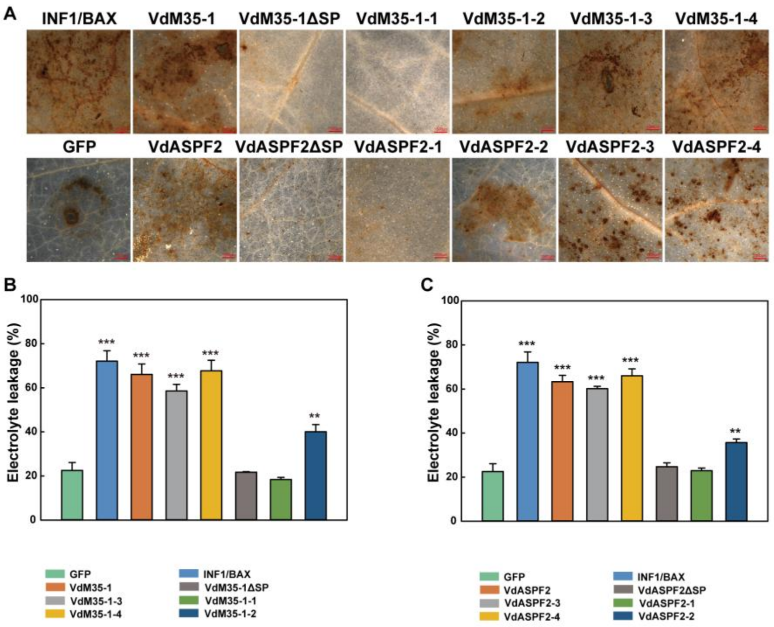

Figure S2: VdM35-1 and VdASPF2 triggered ROS accumulation and electrolyte leakage. (A) ROS accumulation in *N. benthamian* induced by positive control BAX/INF1, negative control GFP, VdM35-1, VdASPF2 and all mutants. (B, C) Analysis of cell death by electrolyte leakage measurements. Values represent means ± standard deviation of three replicates. *, p < 0.05, * *, p < 0.01, * * *, p < 0.001.

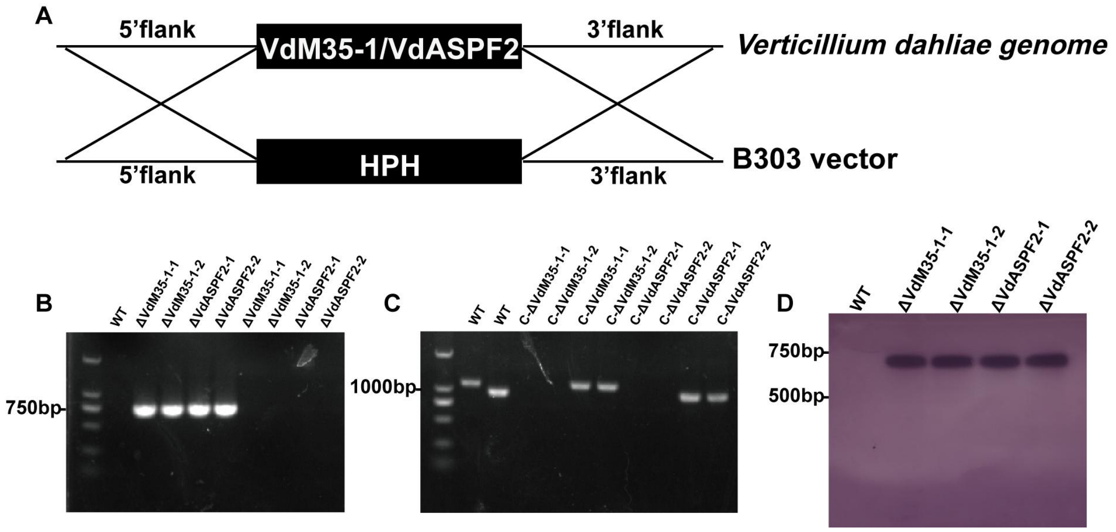

Figure S3: Acquisition of knock-out and complementation mutants. (A) Gene knockout mechanism of *V. dahliae* VdM35-1 and VdASPF2. (B, C) Knockout and complementation strains were determined by PCR. (D) Southern blot was used to identify the knockout strains.

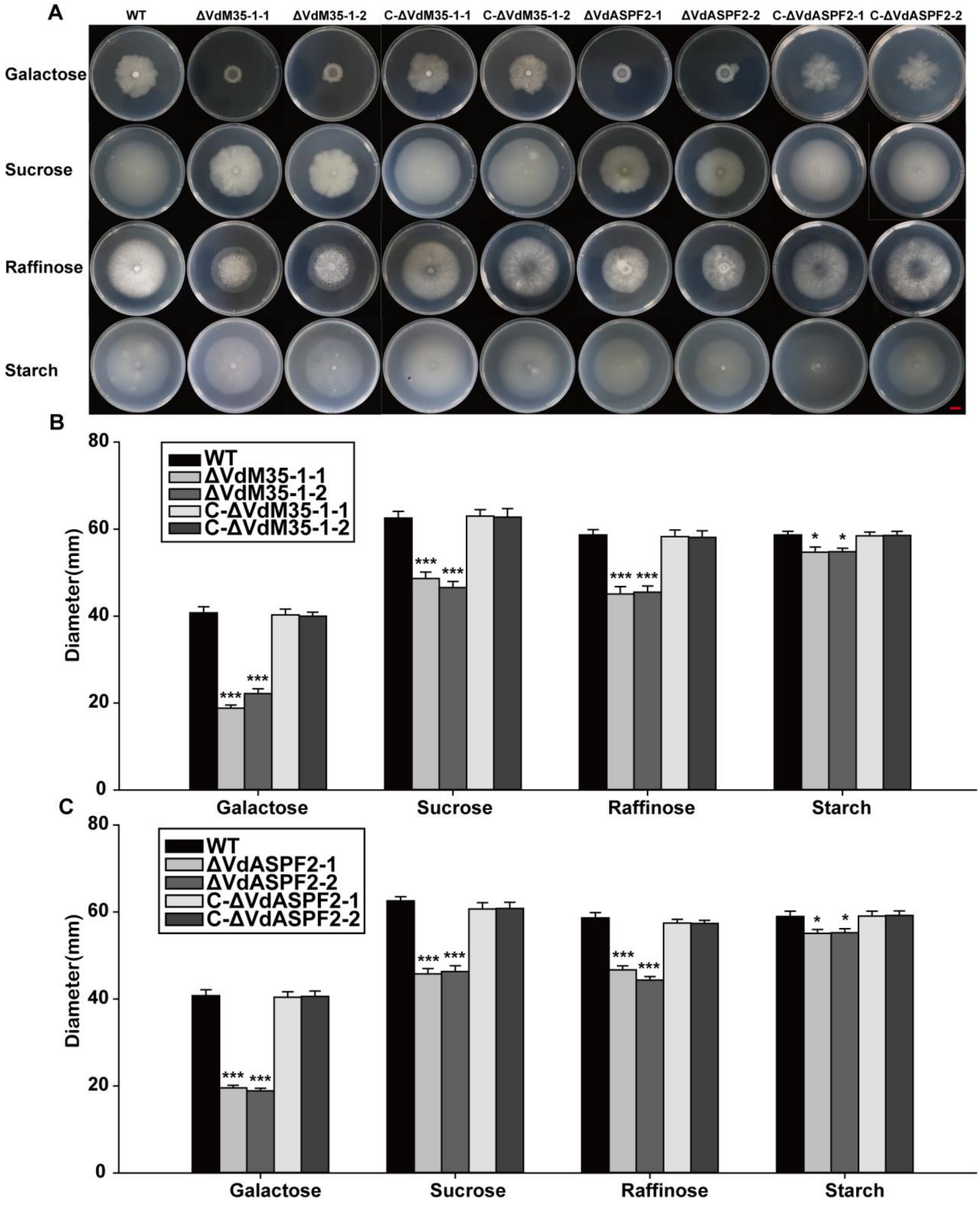

Figure S4: VdM35-1 and VdASPF2 were essential for normal vegetative growth and conidial production of *V. dahliae.* (A) Colony morphology of all strains cultured at 25 °C in dark for 14 days on Czapek-Dox Medium with different carbon sources. Scale = 1 cm (B, C) Colony diameter of all strains. Values represent means ± standard deviation of three replicates. The asterisks represent statistical differences performed by a t-test (*, p < 0.05, * *, p < 0.01, * * *, p < 0.001) in comparison with the wild type strains.

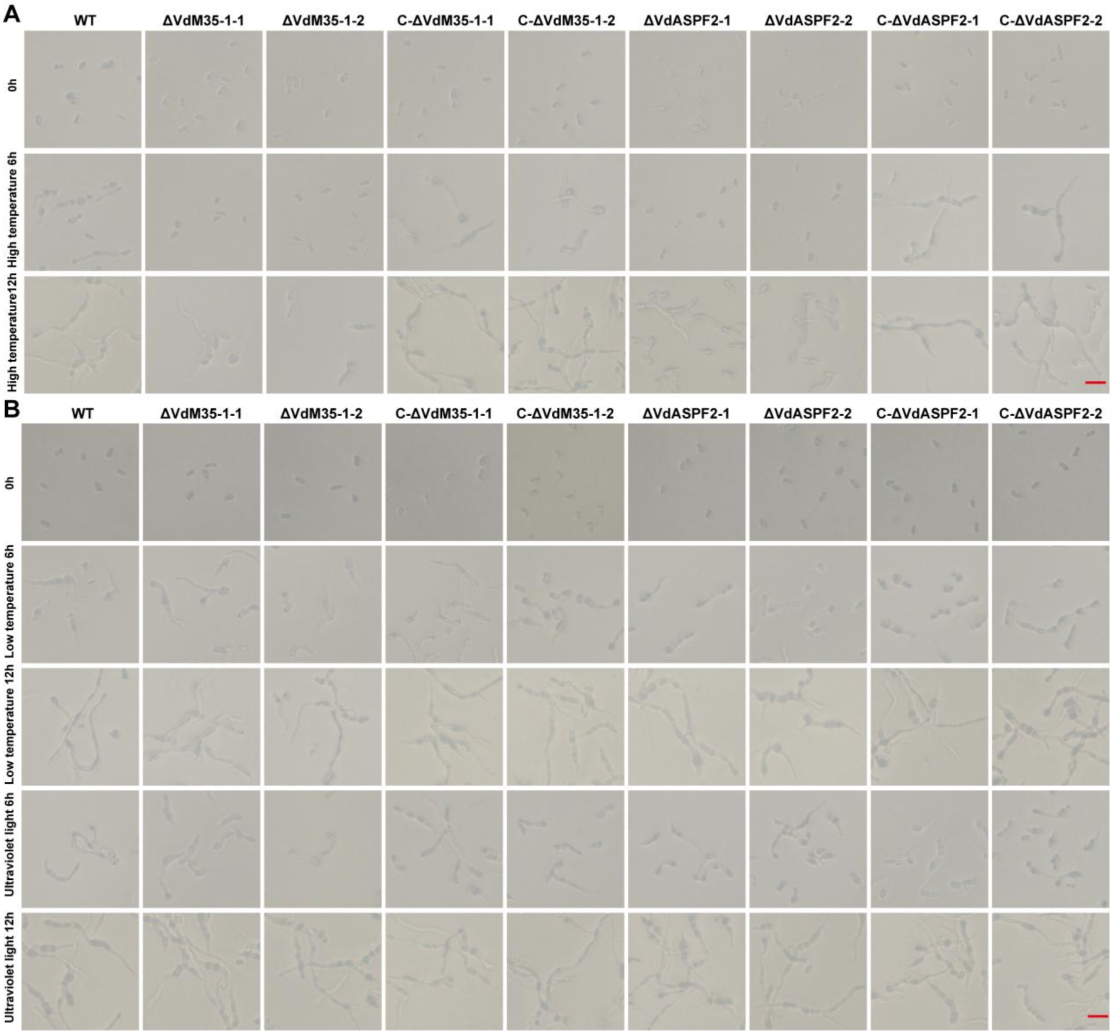

Figure S5: The spore germination of wild, ΔVdM35-1, ΔVdASPF2, C-ΔVdM35-1, and C-ΔVdASPF2 strains under high temperature, low temperature and UV treatment. (A) The conidia of all strains were treated at 45 °C for an hour under fluorescence microscope. (B) The conidia of all strains were treated at 4 °C for an hour and irradiated by ultraviolet light for 3 minutes under fluorescence microscope. Scale = 200μm

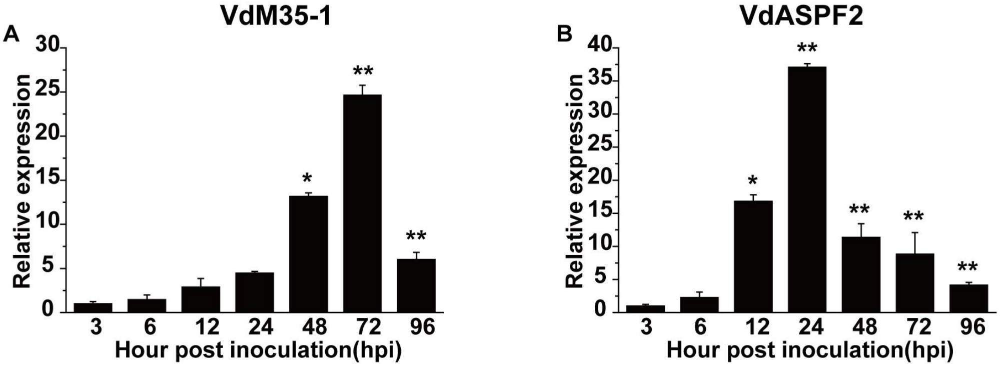

Figure S6: Expression patterns of *VdM35-1* and *VdASPF2*. (A) Expression pattern of *VdM35-1* in cotton infected by *V. dahliae*. (B) Expression pattern of *VdASPF2* in cotton infected by *V. dahliae*. (*, $p < 0.05$, * *, $p < 0.01$)

Table S1. Primers used in this study

Primer name	Sequence (5'-3')	Purpose

VDAG_00129-ClaI-F	GGTCAGCACCAGCTAGCATCGATATGAAGTTTCTCGTGGCC	Clone VDAG_00129 to PGR107 for expression in *N. benthamiana*
VDAG_00129-SmaI-R	TCGCCCTTGCTCACCATCCCGGGGCAACGGGCGAAGATGGA	
VDAG_04551-ClaI-F	GGTCAGCACCAGCTAGCATCGATATGCTTTCTCTCCAGACC	Clone VDAG_04551 to PGR107 for expression in *N. benthamiana*
VDAG_04551-SmaI-R	TCGCCCTTGCTCACCATCCCGGGCGAGCAGTGAACAAAGCCATC	
VdM35-1$^{\triangle SP}$-ClaI-F	GGTCAGCACCAGCTAGCATCGATATGGCCTCGGTCGACTTG	Clone VdM35-1$^{\triangle SP}$ to PGR107for expression in *N. benthamiana*
VdM35-1$^{\triangle SP}$-SmaI-R	TCGCCCTTGCTCACCATCCCGGGGCAACGGGCGAAGATGGA	
NBPR1^{SP}+VdM35-1$^{\triangle SP}$-ClaI-F	GGTCAGCACCAGCTAGCATCGATATGGGATACTCCAAAACATT	Clone NBPR1^{SP}+VdM35-1$^{\triangle SP}$ to PGR107 for expression in *N. benthamiana*
NBPR1^{SP}+VdM35-1$^{\triangle SP}$-R	CAAGTCGACCGAGGCAGCTTGAGATGAGGGAGACA	
NBPR1^{SP}+VdM35-1$^{\triangle SP}$-F	TGTCTCCCTCATCTCAAGCTGCCTCGGTCGACTTG	
VdM35-1-SmaI-R	TCGCCCTTGCTCACCATCCCGGGGCAACGGGCGAAGATGGA	
VdASPF2^{SP}+VdM35-1$^{\triangle SP}$-ClaI-F	GGTCAGCACCAGCTAGCATCGATATGCTTTCTCTCCAGACC	Clone VdASPF2^{SP}+VdM35-1$^{\triangle SP}$ to PGR107 for expression in *N. benthamiana*
VdASPF2^{SP}+VdM35-1$^{\triangle SP}$-R	CAAGTCGACCGAGGCAGAGGCAGCGACGAG	
VdASPF2^{SP}+VdM35-1$^{\triangle SP}$-F	CTCGTCGCTGCCTCTGCCTCGGTCGACTTG	
VdM35-1-SmaI-R	TCGCCCTTGCTCACCATCCCGGGGCAACGGGCGAAGATGGA	
VdASPF2$^{\triangle SP}$-ClaI-F	GGTCAGCACCAGCTAGCATCGATATGCCCGTGGCACGCGCT	Clone VdASPF2$^{\triangle SP}$ to PGR107 for expression in *N. benthamiana*
VdASPF2$^{\triangle SP}$-SmaI-R	TCGCCCTTGCTCACCATCCCGGGCGAGCAGTGAACAAAGCCATC	
NBPR1^{SP}+VdASPF2$^{\triangle SP}$-	GGTCAGCACCAGCTAGCATCGATATGGGATACTCCAAAACA	Clone NBPR1^{SP}+VdASPF2$^{\triangle SP}$ to

ClaI-F	TT	PGR107 for expression in *N. benthamiana*
NBPR1^{SP}+VdASPF2$^{\triangle SP}$-R	AGCGCGTGCCACGGGAGCTTGAGATGAGGGAGACA	
NBPR1^{SP}+VdASPF2$^{\triangle SP}$-F	TGTCTCCCTCATCTCAAGCTCCCGTGGCACGCGCT	
VdASPF2-SmaI-R	TCGCCCTTGCTCACCATCCCGGGCGAGCAGTGAACAAAGCCATC	
VdM35-1^{SP}+VdASPF2$^{\triangle SP}$-ClaI-F	GGTCAGCACCAGCTAGCATCGATATGAAGTTTCTCGTGGCC	Clone VdM35-1^{SP}+VdASPF2$^{\triangle SP}$to PGR107 for expression in *N. benthamiana*
VdM35-1^{SP}+VdASPF2$^{\triangle SP}$-R	AGCGCGTGCCACGGGAGCCCGAGCTAGCAG	
VdM35-1^{SP}+VdASPF2$^{\triangle SP}$-F	CTGCTAGCTCGGGCTCCCGTGGCACGCGCT	
VdASPF2-SmaI-R	TCGCCCTTGCTCACCATCCCGGGCGAGCAGTGAACAAAGCCATC	
VdM35-1$^{\triangle HEXXH}$-ClaI-F1	GGTCAGCACCAGCTAGCATCGATATGAAGTTTCTCGTGGCC	Clone VdM35-1$^{\triangle HEXXH}$ to PGR107 for expression in *N. benthamiana*
VdM35-1$^{\triangle HEXXH}$-R1	CGCAATCTCGGCCAGGAGGATGGTCGTCTG	
VdM35-1$^{\triangle HEXXH}$-F2	CAGACGACCATCCTCCTGGCCGAGATTGCG	
VdM35-1$^{\triangle HEXXH}$-SmaI-R2	TCGCCCTTGCTCACCATCCCGGGGCAACGGGCGAAGATGGA	
VdM35-1$^{\triangle E}$-ClaI-F1	GGTCAGCACCAGCTAGCATCGATATGAAGTTTCTCGTGGCC	Clone VdM35-1$^{\triangle E}$ to PGR107 for expression in *N. benthamiana*
VdM35-1$^{\triangle E}$-R1	GGCCAGATGAGTCATGTGGAGGATGGTCGT	
VdM35-1$^{\triangle E}$-F2	ACGACCATCCTCCACATGACTCATCTGGCC	
VdM35-1$^{\triangle E}$-SmaI-R2	TCGCCCTTGCTCACCATCCCGGGGCAACGGGCGAAGATGGA	
VdASPF2$^{\triangle HRXXH}$-ClaI-F1	GGTCAGCACCAGCTAGCATCGATATGCTTTCTCTCCAGACC	CloneVdASPF2$^{\triangle HRXXH}$ to PGR107 for expression in *N. benthamiana*
VdASPF2$^{\triangle HRXXH}$-R1	GATGCCGGGCAGCAGAAGGTCAGT	
VdASPF2$^{\triangle HRXXH}$-F2	ACTGACCTTCTGCTGCCCGGCATC	
VdASPF2$^{\triangle}$	TCGCCCTTGCTCACCATCCCGGGCGAGCAGTGAACAA	

^{HRXXH}-SmaI-R2	AGCCATC	
VdASPF2$^{\triangle R}$-ClaI-F1	GGTCAGCACCAGCTAGCATCGATATGCTTTCTCTCCAGACC	Clone VdASPF2$^{\triangle R}$ to PGR107 for expression in *N. benthamiana*
VdASPF2$^{\triangle R}$-R1	CAGGTGGAAGGCGTGCAGAAGGTCAGT	
VdASPF2$^{\triangle R}$-F2	ACTGACCTTCTGCACGCCTTCCACCTG	
VdASPF2$^{\triangle R}$-SmaI-R2	TCGCCCTTGCTCACCATCCCGGGCGAGCAGTGAACAAAGCCATC	
VdM35-1SP-EcoR1-F	CCGGAATTCATGAAGTTTCTCGTGGCCAT	Clone VdM35-1 signal peptide to pSUC2 for yeast signal trap assay
VdM35-1SP-XhO1-R	CCGCTCGAGAGCCCGAGCTAGCAGAGCCA	
VdASPF2SP-EcoR1-F	CCGGAATTCATGCTTTCTCTCCAGACCGC	Clone VdASPF2 signal peptide to pSUC2 for yeast signal trap assay
VdASPF2SP-XhO1-R	CCGCTCGAGAGAGGCAGCGACGAGAGGGA	
VdM35-1-Xba1-F	GAGAACACGGGGGACTCTAGAATGAAGTTTCTCGTGGCC	Clone VdASPF2 to p2300 for subcellular localization analysis
VdM35-1-BamH1-R	ACCCATGTTAATTAAGGATCCGCAACGGGCGAAGATGGA	
VdASPF2-Xba1-F	GAGAACACGGGGGACTCTAGAATGCTTTCTCTCCAGACC	Clone VdASPF2 to p2300 for subcellular localization analysis
VdASPF2-BamH1-R	ACCCATGTTAATTAAGGATCCCGAGCAGTGAACAAAGCCATC	
TRV2-BAK1-F	CGGGATCCGTGAGGGTGGTGAGCGGGATAAT	VIGS in *N. benthamiana*
TRV2-BAK1-R	CGGAATTCGCTCATAACTGGGCAAAGGGCTT	
TRV2-SOBIR1-F	CGGGATCCAATCTTTATCCACCAGATCATGC	
TRV2-SOBIR1-R	CGGAATTCCAGAAAGTTTTCCAATGGCAG	
TRV2-GFP-F	CGGGATCCGTGAGCAAGGGCGAGGAGCTGT	
TRV2-GFP-R	CGGAATTCGTCCTTGAAGAAGATGGTGCGCT	
qRT-*NbBAK1*-F	GAGGTGGGAGGAATGGCAAA	qRT-PCR analysis for VIGS efficiency
qRT-*NbBAK*1-R	TTGGCCCCGACAATTCATCT	
qRT-*NbSOBIR1*-F	CCAGCAAGTCACAGAAGGGA	
qRT-*NbSOBIR1*-R	CCAACACCACACCAAAGCTG	
NbActin-qPCR-F	TGGTCGTACCACCGGTATTGTGTT	qRT-PCR analysis in *N. benthamiana*
NbActin-qPCR-R	TCACTTGCCCATCAGGAAGCTCAT	

Nb HIN1-q PCR-F	CCAACTTGAACGGAGCCTATTA	
Nb HIN1-q PCR-R	AGGCATCCAAAGAGACAACTAC	
NbPR1a-qPCR-F	CCGCCTTCCCTCAACTCAAC	
NbPR1a-qPCR-R	GCACAACCAAGACGTACTGAG	
NbPR2-qPCR-F	AGGTGTTTGCTATGGAATGC	
NbPR2-qPCR-R	TCTGTACCCACCATCTTGC	
NbLOX-qPCR-F	AAAACCTATGCCTCAAGAAC	
NbLOX-qPCR-R	ACTGCTGCATAGGCTTTGG	
NbPti5-qPCR-F	CCTCCAAGTTTGAGCTCGGATAGT	
NbPti5-qPCR-R	CCAAGAAATTCTCCATGCACTCTGTC	
NbAcre31-qPCR-F	AATTCGGCCATCGTGATCTTGGTC	
NbAcre31-qPCR-R	GAGAAACTGGGATTGCCTGAAGGA	
NbWRKY7-qPCR-F	CACAAGGGTACAAACAACACAG	
NbWRKY7-qPCR-R	GGTTGCATTTGGTTCATGTAAG	
NbWRKY8-qPCR-F	AACAATGGTGCCAATAATGC	
NbWRKY8-qPCR-R	TGCATATCCTGAGAAACCATT	
Vdβt-F	AACAACAGTCCGATGGATAATT C	
Vdβt-R	GTACCGGGCTCGAGATCG	
VdM35-1-qPCR-F	ACCAGATGCCTCCTACCGACAAG	Expression pattern analysis of *VdM35-1* and *VdASPF2*
VdM35-1-qPCR-R	TCCATAGCCTCCGTAGTCTTCAGTG	
VdASPF2-qPCR-F	GCTCTCTGCGGTGGTGGTTATAC	
VdASPF2-qPCR-R	CGGCGTAATCCTCAGCGTAGTG	
B303-*VdM35-1*-Up-Kpn1-F	GGACCGGACGGGGC<u>GGTACC</u>GGACCGGACGGGGCGGTACC	
B303-*VdM35-1*-Up-R	CTTCAATATCAGTTAACGTCAACAAAATAGGAAACCCGCA	Generation of *VdM35-1* and *VdASPF2* deletion and complementation mutant in *V. dahliae*
B303-*VdM35-1*-HPH-F	TGCGGGTTTCCTATTTTGTTGACGTTAACTGATATTGAAG	
B303-*VdM35-1*-HPH-R	TGCTCTGTTGCCCGCTCCATCTATTCCTTTGCCCTCGGACG	

B303-*VdM35-1*-Down-F	CGTCCGAGGGCAAAGGAATAGATGGAGCGGGCAACAGAGCA	
B303-*VdM35-1*-Down-Pac1-R	TAGTCCCGGGTCTTAATTAACCGAGTCTGAGTCGGTGTTC	
B303-*VdASPF2*-Up-Kpn1-F	GGACCGGACGGGGCGGTACCCGCTATTCATTGTGATGAAA	
B303-*VdASPF2*-Up-R	CTTCAATATCAGTTAACGTCTCACGATGAACCAGGTAGGA	
B303-*VdASPF2*-HPH-F	TCCTACCTGGTTCATCGTGAGACGTTAACTGATATTGAAG	
B303-*VdASPF2*-HPH-R	AAAGGGAGTGCCCACTCACTCTATTCCTTTGCCCTCGGACG	
B303-*VdASPF2*-Down-F	CGTCCGAGGGCAAAGGAATAGAGTGAGTGGGCACTCCCTTT	
B303-*VdASPF2*-Down-Pac1-R	TAGTCCCGGGTCTTAATTAACCGTGAGTGGCTGTGAATGG	
1302-*VdM35-1*-Xba1-F	GACAGCCCGCAAACTCTAGACTAGCTCTATTATAGAGCTA	
1302-*VdM35-1*-Xho1-R	ATTATTATGGAGAAACTCGAGCATAGTACTTTGACGACTCTTCCG	
1302-*VdASPF2*-Xba1-F	GACAGCCCGCAAACTCTAGACGCTATTCATTGTGATGA	
1302-*VdASPF2*-Xho1-R	ATTATTATGGAGAAACTCGAGTCTTCAGCGAGTCGTGA	
VdM35-1-F	ATGAAGTTTCTCGTGGCC	
VdM35-1-R	GCAACGGGCGAAGATGGA	
VdASPF2-F	ATGCTTTCTCTCCAGACC	
VdASPF2-R	CGAGCAGTGAACAAAGCCATC	
HPH-F	ATTTGTGTACGCCCGACAGT	
HPH-R	GTGCTTGACATTGGGGAATT	
HPH-southern-F	ATTTGTGTACGCCCGACAGT	For southern blotting
HPH-southern-R	GTGCTTGACATTGGGGAATT	

Note: Underline for restriction site , and italics for genes or species.
